# (De)sodiation Mechanism of Bi_2_MoO_6_ in Na-Ion Batteries Probed by Quasi-Simultaneous Operando PDF and XAS

**DOI:** 10.1021/acs.chemmater.4c01503

**Published:** 2024-08-02

**Authors:** Anders Brennhagen, Amalie Skurtveit, David S. Wragg, Carmen Cavallo, Anja O. Sjåstad, Alexey Y. Koposov, Helmer Fjellvåg

**Affiliations:** † Centre for Materials Science and Nanotechnology, Department of Chemistry, University of Oslo, P.O. Box 1033, Blindern, Oslo 0315, Norway; ‡ Department of Battery Technology, Institute for Energy Technology, Instituttveien 18, Kjeller 2007, Norway; § FAAM, Strada Statale Via Appia 7 bis, Teverola, Caserta 81030, Italy

## Abstract

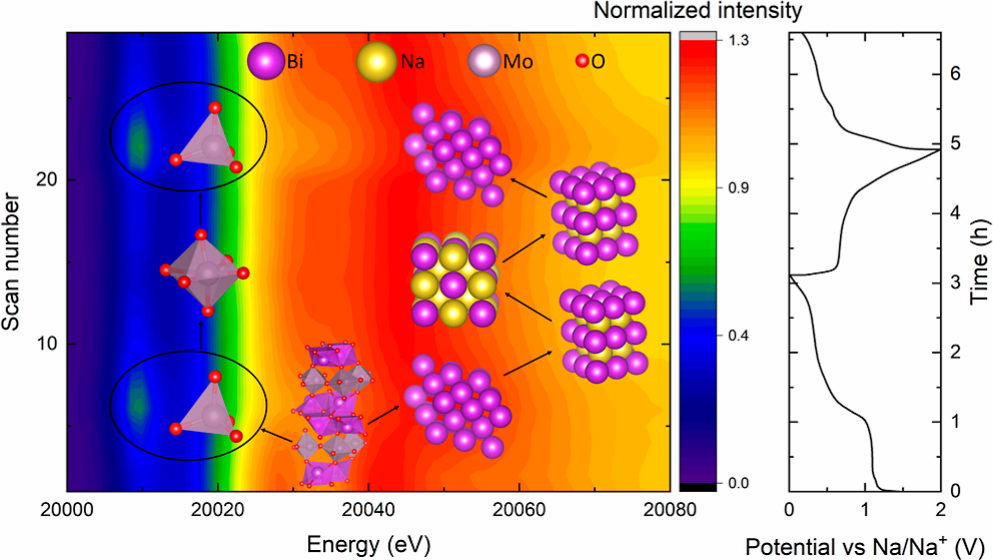

Operando characterization can reveal degradation processes in battery materials and are essential for the development of battery chemistries. This study reports the first use of quasi-simultaneous operando pair distribution function (PDF) and X-ray absorption spectroscopy (XAS) of a battery cell, providing a detailed, atomic-level understanding of the cycling mechanism of Bi_2_MoO_6_ as an anode material for Na-ion batteries. This material cycles via a combined conversion-alloying reaction, where electrochemically active, nanocrystalline Na_*x*_Bi particles embedded in an amorphous Na–Mo–O matrix are formed during the first sodiation. The combination of operando PDF and XAS revealed that Bi obtains a positive oxidation state at the end of desodiation, due to formation of Bi–O bonds at the interface between the Bi particles and the Na–Mo–O matrix. In addition, XAS confirmed that Mo has an average oxidation state of +6 throughout the (de)sodiation process and, thus, does not contribute to the capacity. However, the local environment of Mo^6+^ changes from tetrahedral coordination in the desodiated state to distorted octahedral in the sodiated state. These structural changes are linked to the poor cycling stability of Bi_2_MoO_6_, as flexibility of this matrix allows movement and coalescence of the Na_*x*_Bi particles, which is detrimental to the electrochemical stability.

## 1 Introduction

Na-ion batteries (NIBs) have been demonstrated as a promising alternative to Li-ion batteries (LIBs) in applications such as stationary energy storage, where energy density is less crucial.^1,2^ Despite recent advancements, the development of anode materials with high capacity and stable electrochemical performance represents one of the major challenges for improving modern NIBs. Conversion-alloying materials (CAMs) are among the promising anode candidates, due to their ability to achieve a compromise between high capacity and good cycling stability combined with high-rate capabilities.^3,4^ CAMs operate, in general, by formation of nanoparticles of the alloying element (Si, Ge, Sn, Sb, Bi) inside a stabilizing Na_*x*_X (X = O, S Se, Te, P, oxometallates) matrix through an irreversible conversion reaction during the first sodiation.^4−17^ Subsequently, the alloying (nano)particles account for the capacity during further electrochemical cycling, while the matrix material remains inert. However, some studies reported partially reversible conversion reactions in some of the binary CAMs^7,12^ and electrochemical activity of the transition metal within the matrix in some of the ternary CAMs.^6,16,17^ This indicates the complexity of these chemical systems and illustrates the need for detailed mechanistic studies of these materials under operando conditions.

An appropriate matrix should mitigate the problems associated with the volume expansion of alloying materials, producing CAMs with cycling stability superior to that of pure alloying materials. For example, Bi_2_(MoO_4_)_3_ and BiVO_4_ have shown significantly better cycle life than Bi metal.^16,18^ However, a number of other reported CAMs showed a drastic decay in capacity during the first 10–20 cycles,^19−21,23,24^ raising a question–what controls their cycling stability?

Discrepancies in electrochemical performance for the same active materials are often a result of variations of particle size and morphology, electrode composition and preparation procedures, and electrolyte formulations.^25−27^ However, it has proved difficult to discern general trends regarding how these parameters affect the electrochemical performance.^28^ With these variations, it is difficult to separate the effect of the matrix surrounding the alloying particles from other processes, although it is believed to be one of the most important factors for controlling cyclability in CAMs. Therefore, it is important to understand the formation of the matrix and its chemical evolution during cycling for the rational design of CAMs.

The amorphous nature of most matrixes formed in CAMs limits the useful information that is possible to extract from the most commonly used operando technique for structural characterization of battery active materials—X-ray diffraction (XRD).^9,29,30^ Instead, X-ray pair distribution function (PDF) analysis, which provides local structural information on both amorphous and crystalline materials, can be used.^31,32^ Examples of operando PDF studies on active materials for LIBs^15,30,33−38^ and NIBs^9,39−42^ are few, and to the best of our knowledge such analysis have not been previously used to study CAMs for NIBs. This can be attributed to the difficulty of removing nonsample contributions from operando total scattering data collected from batteries prior to obtaining the PDF.^32^ However, one recent study by Sottmann et al. using operando total scattering computed tomography (TSCT), combining XRD and PDF analysis, on BiVO_4_ as anode material in LIBs brought the understanding of the cycling mechanism of CAMs one step further by providing clear structural information on the amorphous components of the cycling process.^15^

Bi metallates are a group of ternary CAMs, of which BiFeO_3_, Bi_2_(MoO_4_)_3_, BiVO_4_ and Bi_2_MoO_6_ have previously been electrochemically evaluated in half cells for NIBs with varying success in stabilizing the materials’ capacity.^8,16,21−23,43^ Like other CAMs, Bi metallates undergo an irreversible conversion reaction during the first sodiation: Bi (nano)particles become embedded in a Na–TM–O matrix (TM = transition metal). The Bi particles can then reversibly alloy with Na^+^ to form cubic Na_3_Bi (c-Na_3_Bi) through NaBi as an intermediate.^16,23^ The dramatic variation in performance of Bi metalates has shifted the research focus toward detailed understanding of the cycling mechanism. However, the structural evolution of Bi particles and Na–TM–O matrix during electrochemical cycling has proven to be difficult to characterize. A study combining operando XRD, X-ray absorption near edge spectroscopy (XANES) and density functional theory calculation studies of Bi_2_(MoO_4_)_3_ suggested that the matrix had an average chemical formula of Na_2_MoO_4_ in the desodiated state, which transformed into Na_3_MoO_4_ in the sodiated state.^16^ Another study, of Bi_2_MoO_6_, suggested a further conversion to Mo metal and Na_2_O.^22^ Using operando X-ray absorption spectroscopy (XAS), Surendran et al. reported the formation of small amounts of metallic Fe together with Na_2_O and an undefined Fe–O phase when BiFeO_3_ was studied as a potential anode material in NIBs.^8^ The same study demonstrated the appearance of Bi–O bonds within the active material after the first desodiation, indicating partial oxidation of Bi metal.^8^ These results indicate that the Na–TM–O matrix, which ideally should be inert, can participate in the electrochemical transformations.

Our previous studies on Bi_2_MoO_6_^23^ and BiFeO_3_^24^ indicated that the growth of the Na_*x*_Bi (0 ≤ *x* ≤ 3) (nano)particles within the Na–TM–O matrix, probably through electrochemical sintering,^44^ is a likely reason for the limited cycling stability of these materials. This growth could be due to structural changes in the matrix that allows movement and coalescence of the Na_*x*_Bi particles. The chemical nature of the interaction between the Na–TM–O matrix and Na_*x*_Bi (nano)particles remained unknown. In this work we address this problem by using quasi-simultaneous operando PDF/XAS to obtain an understanding of the (de)sodiation mechanisms of Bi_2_MoO_6_, with a particular focus on structural changes within the Na–Mo–O matrix and its interactions with Na_*x*_Bi (nano)particles. The operando PDF, XRD and Fourier transformed (FT) extended X-ray absorption fine structure (EXAFS) data provide a full overview of the local and long-range structures forming in the sample, while the XANES data are used to track the oxidation states and local coordination of both Bi and Mo during (de)sodiation. By this unprecedented combination of techniques, we can accurately describe the electrochemical processes occurring in Bi_2_MoO_6_ during (de)sodiation in much greater detail than previously. The results provide a deeper understanding of the interplay between the alloying particles and the matrix and how this effects the cycling performance.

## 2 Experimental Section

### 2.1 Chemicals

Bi(NO_3_)_3_·5H_2_O (98%), Bi_2_O_3_ (99.8%), Bi powder (99%), Bi foil (8 μm thick, 99.97%), Na_2_MoO_4_ (anhydrous, 99.9%), Mo powder (99.9%), MoO_2_ (99%), MoO_3_ (99.97%), ethylene glycol (99.8%), diethyl carbonate (DEC, 99%), *N*-methyl-2-pyrrolidone (NMP, 99.5%), ethylene carbonate (EC, 99%), fluoroethylene carbonate (FEC, 99%) and Na_2_MoO_4_·2H_2_O (99%) were purchased from Sigma-Aldrich. Ethanol (99.7%) was bought from VWR, Super P from Timical, Kynar polyvinylidene fluoride (PVDF) from Arkema, double-sided dendritic Cu foil (99.9%, 10 μm thick) from Schlenk and NaPF_6_ from Fluorochem. Na, DEC, EC, PC, FEC and NaPF_6_ were stored in an Ar-filled glovebox (MBraun Labmaster, H_2_O and O_2_ < 0.1 ppm), while ethylene glycol and NMP had protective caps. All chemicals were used as purchased without any further purification or treatments unless specified in the following sections.

### 2.2 Synthesis of Bi_2_MoO_6_

The solvothermal synthesis of Bi_2_MoO_6_ was adopted from our previous study with small modifications.^23^ 5.2 mmol (1.26 g) Na_2_MoO_4_·2H_2_O and 10.4 mmol (5.06 g) Bi(NO_3_)_3_·5H_2_O were each dissolved in 15 mL of ethylene glycol in two separate beakers. After stirring for half an hour, the solutions were mixed in a third container and 60 mL of ethanol was added dropwise at a rate of approximately two drops per second. The resulting mixture was stirred for 1 h and transferred to a 180 mL Teflon-lined stainless steel autoclave, which was heated at 200 °C for 6 h in a furnace. The autoclave was naturally cooled down to room temperature. The resulting Bi_2_MoO_6_ particles were collected by filtration, washed 3 times with ethanol and dried overnight at 60 °C in air.

### 2.3 Electrode Preparation

Electrodes containing Bi_2_MoO_6_ were prepared by mixing 80 wt % active material (Bi_2_MoO_6_) with 10 wt % Super P and 10 wt % PVDF binder in NMP in a Thinky mixer (ARE 250). The total dry mass for each batch was 1 g and the amount of NMP was 4 mL. The mixing program consisted of 2 rounds of mixing at 2000 rpm for 3 min followed by one defoaming step at 700 rpm for 2 min. The resulting slurry was coated on 10 μm thick double-sided dendritic Cu foil as current collector using a stainless steel coating bar with a fixed height of 300 μm for coin cells and 1000 μm for the operando cell. The thicker electrode for the operando measurement was necessary to obtain sufficient intensity of the X-ray signals. The electrode sheets were dried overnight in a fume hood under ambient conditions before they were cut into discs of 15 mm in diameter. Then, the electrodes were dried in a Buchi oven at 80 °C under dynamic vacuum for 4 h before being inertly transferred to an Ar-filled glovebox (MBraun Labmaster, H_2_O and O_2_ < 0.1 ppm). The active mass loadings of the electrodes were ∼2–4 and ∼5–7 mg cm^–2^ for the 300 and 1000 μm coatings, respectively.

### 2.4 Electrochemical Analysis

The electrochemical performance of Bi_2_MoO_6_ was evaluated in half cells with Na metal as a counter electrode. The coin cells had CR2032 stainless steel housings (304, Pi-Kem) and glass microfiber separators (16 mm, Whatman grade GF/C). During cell assembly, the separator was soaked in 80 μL of electrolyte: 1 M NaPF_6_ in PC with 5% FEC. Na metal electrodes were prepared from Na blocks (Sigma-Aldrich) by removing the oxide layer with a scalpel, rolling the blocks into thin sheets (∼0.5 mm thick) and punching out discs of 14 mm in diameter. An automatic coin cell crimper (Hohsen) pressed and sealed the cells. Galvanostatic cycling (GC) was performed with various cutoff voltages, in the range of 0.01–2.00 V vs Na/Na^+^, and specific currents between 0.02 and 1.00 A g^–1^ by using a battery tester from Neware (MIHW-200-160CH).

### 2.5 Preparation of Post-Mortem Samples for XRD and XAS

Electrodes for post-mortem XRD and XAS were prepared with the procedure described in Sections 2.3 and 2.4. The batteries were cycled at 0.1 A g^–1^ between 0.01 and 2.00 V vs Na/Na^+^ until the desired stage of cycling (specified in the corresponding figures) where the voltage was held constant until the current was lower than 20 μA. The cells were disassembled with a coin cell disassembling tool (Hoshen) inside the Ar-filled glovebox. The electrodes were carefully extracted from the disassembled batteries, cleaned with ∼0.5 mL DEC per electrode and dried inside the glovebox for 1 h. Following this, the electrode material was scraped off from the Cu foil, ground carefully in a mortar and packed in 1 mm borosilicate glass capillaries sealed with UV glue.

### 2.6 Ex Situ X-Ray Characterization

The XRD/PDF and XAS measurements were performed at beamline BM31, which is a part of the Swiss-Norwegian Beamlines at the European Synchrotron Radiation Facility (ESRF). The reference samples were diluted with 20 wt % carbon black (Super P) to increase the data quality of the XAS measurements. All samples were packed in 1 mm borosilicate glass capillaries (Hilgenberg) and sealed with UV glue (Bondic) in an Ar-filled glovebox (MBraun Labmaster, H_2_O and O_2_ < 0.1 ppm). A Pilatus CdTe 2 M detector from DECTRIS and monochromatic synchrotron radiation with a wavelength of 0.24486 Å (*E* = 50.6338 keV) were used for the XRD/PDF measurements with 15 repetitions of 20 s exposure resulting in a total time of 5 min per scan. XAS measurements were performed in transmission mode on the Bi L3 edge with ion chamber detectors in the energy range of 13.32–14.10 keV, 0.7 eV step size and 200 ms exposure resulting in a measurement time of 3 min 44 s per scan. The Mo K edge was measured similarly with an energy range of 19.9–20.8 keV, 0.8 eV step size and 200 ms exposure leading to a measurement time of 3 min 21 s per scan. For the XAS measurements, 3 scans for each ex situ sample were performed resulting in a total measurement time of 11 min 12 s for each Bi measurement and 10 min 3 s for Mo. The scans were averaged and analyzed in Athena.^45^

### 2.7 Operando XRD/PDF/XAS Measurement

The operando cell, a piston-type cell with glassy carbon as X-ray windows, has been described in detail in earlier publications.^23,46^ An MPG2 battery cycler from Biologic performed the GC measurement between 0.01 and 2.00 V vs Na/Na^+^. Due to limited beamtime a specific current of 0.2 A g^–1^ was used instead of 0.1 A g^–1^ as for the ex situ samples. This difference in applied current density is expected to have minimal impact on the results as the electrochemical performance is very similar at these two current densities. The electrochemical cycling ran continuously while the alternating XRD/PDF and XAS measurements were performed. The combined operando measurement was performed with one scan for each measurement resulting in a total measurement time of 10 min 44 s per sequence. The details of the XRD/PDF and XAS measurements are described in Section 2.6.

### 2.8 Data Processing

The 2D diffraction data were gain corrected (correcting for nonuniform response in the pixels of the detector), normalized to the varying intensity of the beam, averaged, and integrated with Python scripts provided by the BM31 staff.^47^ For the operando measurement, the background (empty operando cell) was subtracted in pdfgetx3 (through pdfgetx3_gui.py^47^) with a background scale of 1. Cu and Na peaks from the operando measurement were subtracted using a homemade Python script (peakremoval_xy.py^48^), where start and end values in *Q* were chosen individually for the diffraction peaks. Different start and end *Q* values were used for scans 0–4, because of the presence of Bi_2_MoO_6_ peaks, and scans 5–29 (Section S3, Supporting Information). To convert the peak- and background-subtracted XRD patterns to PDFs (*G*(*r*)) we used pdfgetx3_gui.py with a background scale of 0, *Q*_min_ = 0.50, *Q*_max_ = 15, *Q*_maxinst_ = 22, *r*_poly_ = 0.98.

The XRD data from ex situ measurements were converted to PDFs using pdfgetx3_gui.py by manually adjusting the background scale (for appropriate subtraction of the Ar-filled 1 mm capillary background) and with the same *Q* and *r*_poly_ values as for the operando data. Rietveld refinements and fitting of PDF data were performed with Topas v6.^49^

Athena was used for processing the XAS data.^45^ For the operando measurement, there were some consistent noise peaks at energies slightly below the Mo K edge that were removed before further processing. These peaks were negligible for the ex situ samples and removal was not necessary. The edge position was chosen as the position of the maximum value of the first peak in the first derivative of the XANES spectra for Bi and the second peak for Mo (as the first peak corresponded to the pre-edge). A spline range in *k* of 0–13 was used with a *k* range of 3–11 for the Fourier transform. For the Mo edge we set *R*_bkg_ = 1.0 and *R* range for backward Fourier transform to 1–6. The *R*_bkg_ values for the Bi edge were set to 1.23 for the operando data and 1.21 for ex situ samples. An *R* range of 1–5 was used for backward Fourier transform for all the Bi L3 measurements. Linear combination fitting (LCF) was performed on Bi L3 XANES data measured on the fully desodiated samples by using Bi metal foil and Bi_2_O_3_ as references to obtain an estimation of the average oxidation state.

## 3 Results and Discussion

### 3.1 Materials and Electrochemical Characterization

The synthesized material was orthorhombic Bi_2_MoO_6_ (COD: 1530868) as confirmed by XRD/PDF (Figure S1, Supporting Information). The sample was shown to have the expected average oxidation states of +3 for Bi and +6 for Mo in Bi_2_MoO_6_ by analysis of the XANES edge positions, using Bi_2_O_3_, Na_2_MoO_4_ and MoO_3_ as references (Figure S2, Supporting Information). The low pre-edge feature in the XANES spectra of the Mo K edge is characteristic of the distorted octahedral coordination of Mo^6+^ in Bi_2_MoO_6_ (similar to that of Mo^6+^ in MoO_3_), as opposed to the tetrahedrally coordinated Mo^6+^ in Na_2_MoO_4_ (Figure S2c, Supporting Information).

**Figure 1:**
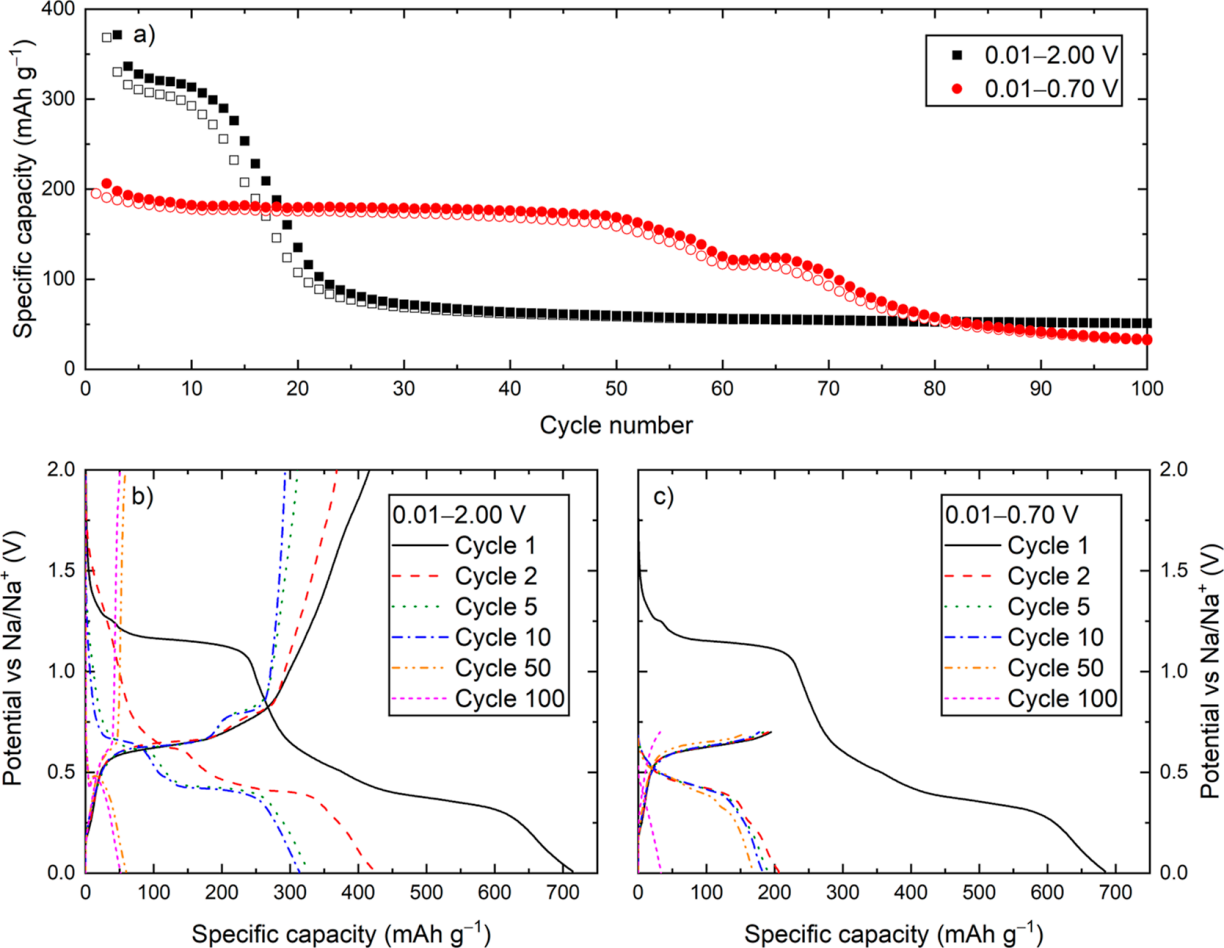
(a) Specific capacity as a function of cycle number extracted from GC measurements of Bi_2_MoO_6_ cycled with a specific current density of 0.1 A g^–1^ and voltage ranges of 0.01–2.00 V (black symbols) and 0.01–0.70 (red symbols) vs Na/Na^+^. Filled symbols represent sodiation and open symbols represent desodiation capacities. (b,c) corresponding (de)sodiation curves.

The electrochemical performance of Bi_2_MoO_6_, when cycled with a current density of 0.1 A g^–1^ and a voltage window of 0.01–2.00 V vs Na/Na^+^, was similar to that reported in our previous study.^23^ The specific capacity of the first sodiation is ∼700 mAh g^–1^, which was reduced to ∼400 mAh g^–1^ in the second sodiation mainly because of the loss of the initial irreversible conversion reaction (Figure 1b). The capacity was maintained between 300 and 400 mAh g^–1^ for ∼10 cycles, before it rapidly decayed to <100 mAh g^–1^ after 20 cycles (Figure 1). Varying the current densities from 0.02–1.00 A g^–1^ had minimal impact on the performance except for increasing overpotential with higher current densities (Figure S4, Supporting Information). Previous studies of BiFeO_3_ achieved significantly increased cycling stability by reducing the upper cutoff voltage to 0.70 V, thus isolating the NaBi–Na_3_Bi reaction.^24^ Applying the same strategy to Bi_2_MoO_6_ gave a similar improvement, with capacity close to 200 mAh g^–1^ after 50 cycles in the voltage range of 0.01–0.70 V vs Na/Na^+^ compared to ∼50 mAh g^–1^ when cycled between 0.01 and 2.00 V vs Na/Na^+^ (Figures 1 and S5, Supporting Information). This change in cycling performance at the two different voltage windows could be linked to different chemical activity of the matrix surrounding the alloying (nano)particles.

### 3.2 Operando XANES

**Figure 2:**
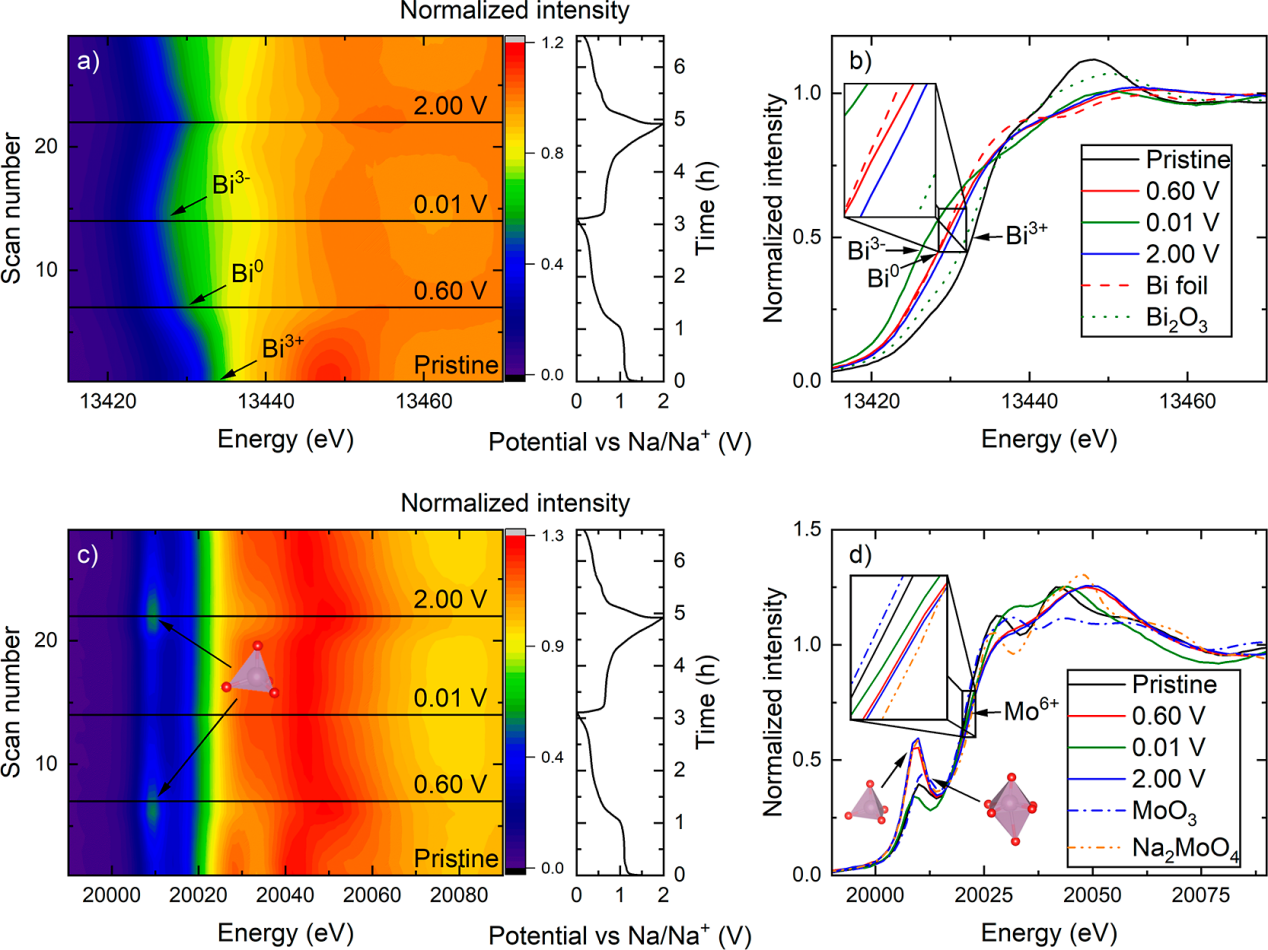
Contour plots obtained from operando XANES measurement of (a) Bi L3 edge and (c) Mo K edge, including corresponding (de)sodiation curves obtained from GC at 0.2 A g^–1^ and 0.01–2.00 V vs Na/Na^+^. Selected scans of (b) Bi L3 edge and (d) Mo K edge vs references with zoomed in insets to highlight the differences in edge position. The black lines in (a,c) correspond to the XANES graphs shown in (b,d).

In order to monitor the structural and electronic changes in the matrix and its interaction with the Na_*x*_Bi particles we performed an operando measurement combining X-ray total scattering (XRD and PDF) with XAS (XANES and EXAFS). The use of XAS allows estimation of the changes in the oxidation state and coordination of Bi and Mo during cycling. This information links the chemical transformations of the active component (Bi) to those of the main matrix component (Mo). The XRD data from the first 1.5 (de)sodiation cycles show the expected transformations between the (nano)crystalline phases: Bi_2_MoO_6_ → Bi ⇋ NaBi ⇋ c-Na_3_Bi (Figure S7, Supporting Information). Operando XANES confirms these processes via drastic changes in the Bi-oxidation state (Figure 2a,b). After the initial conversion reaction, completed at 0.60 V vs Na/Na^+^, the Bi L3 edge position overlapped with the Bi-foil reference, indicating that the average oxidation state of Bi changed from +3 to 0 (Figure 2b). The oxidation state of Bi then gradually decreased from 0 until it reached −3 by the end of sodiation (0.01 V vs Na/Na^+^), when c-Na_3_Bi has formed. During the subsequent (first) desodiation, the average oxidation state of Bi increased as expected, but reached a value larger than 0 in the fully desodiated state (at 2.00 V vs Na/Na^+^) as the final edge position in the XANES spectra was at a higher absorption energy than that of the Bi-metal reference. The positive oxidation state for Bi at the end of desodiation has previously been observed for BiFeO_3_,^8,24^ but was not reported for other Bi metallates.^16,22,23^

The XANES Mo K edge position can serve as an indicator for the electrochemical participation of Mo. There is a tiny shift of the Mo K edge position during cycling, however, the edge position stays in between the positions of the Mo^6+^ references (Na_2_MoO_4_ and MoO_3_), implying that the oxidation state of Mo remains +6 throughout the electrochemical cycling (Figure 2c,d) and that the shift in edge position is rather an effect of change in coordination. The pre-edge feature of the Mo K edge spectra increases in intensity during the initial conversion reaction, indicating a change of Mo^6+^ coordination from distorted octahedral to tetrahedral, as confirmed by comparison with the Na_2_MoO_4_ reference that contains tetrahedrally coordinated Mo^6+^. During further sodiation, 0.60–0.01 V vs Na/Na^+^, Mo^6+^ reverts to an almost octahedral coordination. This last step is reversed during desodiation when Mo^6+^ becomes tetrahedrally coordinated in the fully desodiated state at 2.00 V vs Na/Na^+^. These structural changes in the matrix, shown by the pre-edge peak, could be linked to the poor cycling stability of Bi_2_MoO_6_ when cycling across the full voltage range (0.01–2.00 V vs Na/Na^+^), as will be discussed later.

### 3.3 Operando FT EXAFS and PDF

**Figure 3:**
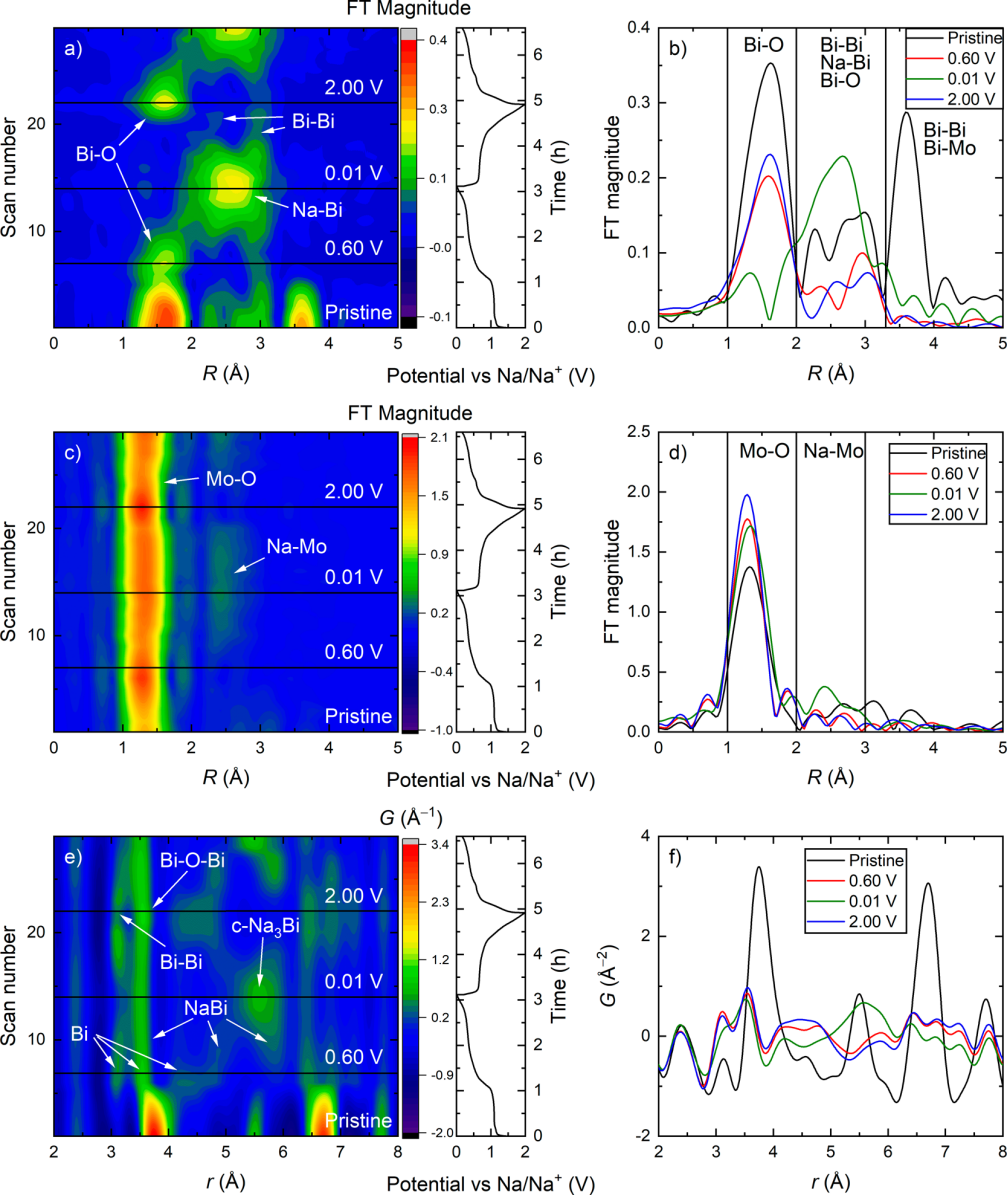
FT EXAFS contour plots of (a) Bi L3 and (c) Mo K, including (de)sodiation curves obtained by GC at 0.2 A g^–1^ and 0.01–2.00 V vs Na/Na^+^. (e) PDF contour plot with labels highlighting the peaks corresponding to the closest Bi–Bi bonds in each of the Na_*x*_Bi phases. (b), (d,f) selected scans extracted from the plots in (a,c,e) marked with black lines.

The operando FT EXAFS and PDF data provided additional information on changes in the local structure of the active material during (de)sodiation (Figure 3). Bi-L3 FT EXAFS graphs of the initial conversion reaction (Figure 3a,b) illustrate disappearance of the peaks above 3.3 Å, which correspond to longer range Bi–Bi and Bi–Mo distances present in the original Bi_2_MoO_6_ material, in the initial sodiation (until 0.60 V vs Na/Na^+^). This confirms the loss of crystallinity during the initial conversion reaction. The Bi-L3 EXAFS peaks for Bi–Bi and Na–Bi bonds are found in the region between 2.0 and 3.3 Å, and overlap makes them difficult to distinguish from one another. However, we attribute the large peak centered at ∼2.6 Å present at 0.01 V to Na–Bi bonds from the c-Na_3_Bi phase (Figure 3a,b), which clearly dominates the XRD data at this stage of cycling (Figure S7, Supporting Information). Likewise, the strong double peak observed at ∼3.0 Å in the EXAFS spectrum collected at 0.60 V vs Na/Na^+^ can only be due to Bi–Bi bonds. The intensity of the peak corresponding to Bi–O bonds (1–2 Å) declines through the conversion reaction but does not disappear until the start of NaBi phase formation, demonstrating that Bi–O bonds coexist with Bi-metal (nano)particles. During desodiation, the EXAFS (Figure 3a,b) signals of Na–Bi bonds decrease together with an increase of the signals related to Bi–Bi bonds as the system transforms back to Bi metal (also seen in the XRD, Figure S7, Supporting Information). Toward the end of desodiation (at 2.00 V vs Na/Na^+^), the Bi–O bond FT EXAFS peaks reappear as the XANES data show Bi obtaining an average positive oxidation state (Figure 3a,b). At this state of charge, the presence of Bi metal is confirmed by both XRD and FT EXAFS, and the Bi–O bonds can be rationalized through the interaction between the Bi (nano)particles and the Na–Mo–O matrix at the interface.

As mentioned above, the oxidation state of Mo does not change through the electrochemical cycling, however, the transition of Mo^6+^ from distorted octahedral coordination (pristine) to tetrahedral (0.60 V vs Na/Na^+^) is noticeable. Close examination of the FT EXAFS plots (Figure 3c,d) for Mo reveals that the peak corresponding to the closest Mo–O bonds becomes sharper, more intense and shifts slightly toward lower *R* values during the change of coordination. This sharper peak corresponds to the expected Mo–O bond length of tetrahedrally coordinated Mo^6+^ (1.787 Å) in Na_2_MoO_4_, rather than those of the distorted octahedra in Bi_2_MoO_6_ (six different Mo–O bonds between 1.779 and 2.296 Å). At a later stage of sodiation (∼0.4 V vs Na/Na^+^) the Mo–O peak becomes broader and shifts toward higher *R* values, confirming that the coordination switches back to distorted octahedral, together with the appearance of a peak at ∼2.5 Å (Figure 3c,d). It is not clear what type of scattering events that give rise to this peak, but fitting of the FT EXAFS data from a fully sodiated ex situ sample indicated that it corresponds to Na–Mo interactions (with atomic distances in the range of 2.8–3.3 Å) similar to those reported for the crystal structure of Na_4_MoO_5_ (Section S10, Supporting Information). The structure of the Na–Mo–O matrix with distorted octahedral coordination for Mo^6+^ seems to be maintained below 0.70 V vs Na/Na^+^ during desodiation, and first switches back to the tetrahedral configuration at higher voltages. This explains the increased cycling stability when the Bi_2_MoO_6_-based electrodes were cycled between 0.01 and 0.70 V vs Na/Na^+^ (Figure 1), since a structurally stable matrix could prevent movement and coalescence of the alloying particles, thereby, stabilizing the performance (see further justification in Section 3.5).

The peaks at *r* > 30 Å in the operando PDFs disappear during the initial conversion reaction confirming loss of long-range order (Figure S8, Supporting Information). In addition, the data show that there are significant changes in the local structure (*r* < 8 Å) of the material in the following cycling (Figure 3e,f). Due to the large difference in atomic number of the studied elements (Bi vs Mo, Na and O), the PDFs are dominated by the signals corresponding to the atomic distances between Bi atoms. The main features of the PDF data correspond well to the results from XRD and XAS, and the presence of metallic Bi (0.60 V vs Na/Na^+^) was confirmed by PDF peaks at ∼3, ∼3.5 and ∼4.6 Å (Figure 3e) corresponding to the 4 closest Bi–Bi bonds of Bi metal (theoretical values: 3.07, 3.53, 4.55, and 4.75 Å, Table S2, Supporting Information). The closest Bi–Bi bonds in NaBi are visible through peaks at ∼3.5, ∼4.8 and ∼5.9 Å, while the closest Bi–Bi bonds in c-Na_3_Bi correspond to the peak at ∼5.5 Å (theoretical: 5.42 Å).

The PDF peak at ∼3 Å, corresponding to the closest Bi–Bi bonds in Bi metal (Figure 3e,f), is present through most of the sodiation until the final transformation to c-Na_3_Bi, even in the region where NaBi is expected to be the dominant phase based on the signals above 4 Å. One explanation for the persistence of the peak at ∼3 Å is that nanosized Bi clusters, similar to those observed in operando TSCT of BiVO_4_ for LIBs,^15^ coexist with other clusters that have transformed to NaBi. Another possible explanation is that some Na–Bi bonds are shorter than the expected 3.43 Å, as the disordered and dynamic nature of the nanosized clusters may allow bonding possibilities that are not expected to exist in more crystalline systems. However, given the intensity of the peak at ∼3 Å, it most likely corresponds to Bi–Bi bonds and not Na–Bi, meaning that the shortest bonds of Bi metal are kept long after the Bi-metal interactions at >4 Å disappears. This peak, at ∼3 Å, shifts toward higher *r* values during sodiation before it disappears, showing that the bond length increases until the Bi–Bi bonds finally break during formation of c-Na_3_Bi.

During desodiation, the peak at ∼3 Å reappears showing clear Bi–Bi bonds throughout most of the desodiation (Figure 3e,f). However, toward the end of desodiation the peak’s intensity decreases together with an increase in intensity of the peak at ∼3.5 Å. This change is explained by the oxidation of metallic Bi, as was shown in the XAS data, forming Bi–O bonds at the interface toward the Na–Mo–O matrix: the Bi–Bi bonds at ∼3 Å are replaced with Bi–O–Bi at ∼3.5 Å, similar to the closest Bi–Bi distances in Bi_2_O_3_ (Table S2, Supporting Information). At the beginning of the second sodiation, the peak at ∼3 Å regains intensity as the Bi–O interactions diminished before the formation of NaBi.

### 3.4 Particle Growth and Capacity Degradation

**Figure 4:**
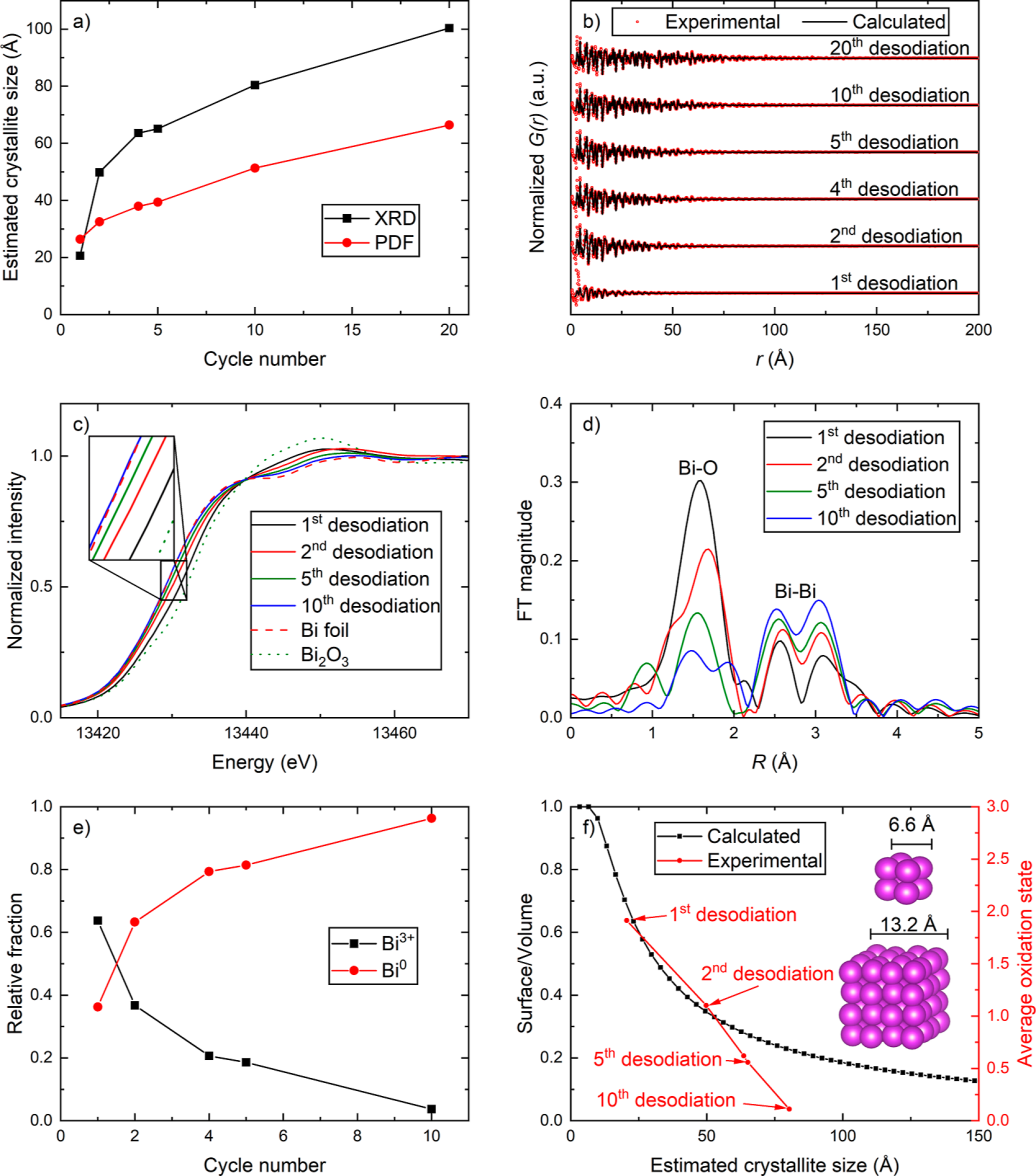
(a) Estimated average sizes of the Bi crystallites in desodiated Bi_2_MoO_6_ samples as a function of cycle number. The sizes were estimated with 2 different methods. Black line represents LVol-IB (integral breadth) calculated from CS_G (crystallite size Gaussian), which was obtained from Rietveld refinements of XRD data in Topas.^49^ The red line represents spherical damping values obtained from fitting the corresponding PDF data. (b) PDFs of desodiated Bi_2_MoO_6_ samples with fittings that were used to create the red graph in (a). (c) Ex situ XANES of Bi_2_MoO_6_ samples desodiated to 2.00 V from different cycles. (d) Corresponding FT EXAFS graphs indicating Bi–O and Bi–Bi bonds. (e) Relative fractions of Bi^3+^ vs Bi^0^ obtained from LCF of the XANES spectra shown in (c) by using Bi_2_O_3_ as a reference for Bi^3+^ and Bi metal foil as a reference for Bi^0^. (f) Estimated surface-to-volume ratios and average oxidation states of Bi particles as a function of particle size assuming cubic particles with primitive packing of Bi atoms with 3.3 Å in diameter where the surface atoms have an oxidation state of +3. Black curve shows the theoretical calculations while the red curve shows the estimated oxidation states based on the LCF in (e) and the crystallite sizes estimated from XRD data shown in (a) based on experimental data of the desodiated ex situ samples.

The capacity degradation during the first 20 cycles for Bi_2_MoO_6_ and BiFeO_3_ has previously received considerable attention, where growth of the alloying particles was suggested to be the main driver of electrochemical deactivation.^23,24^ To provide experimental evidence for this hypothesis, a set of ex situ samples was analyzed to determine the size of Bi (nano)particles. Ex situ XRD and PDF data from fully desodiated (2.00 V vs Na/Na^+^) samples extracted after 1st, 2nd, 5th and 10th desodiation, confirmed that the average crystallite sizes of Bi, and thus the size of the alloying particles, increased significantly during cycling (Figure 4a,b). The estimations obtained from XRD and PDF are slightly different but showed the same trend, where the average size of the Bi crystallites increased from 2 to 3 nm after the first desodiation to 6–10 nm after the 20th desodiation (Figure 4a). This growth leads to larger distances between the Na_*x*_Bi particles, thus creating challenges for the transport of electrons through the insulating Na–Mo–O matrix and Na^+^ through the sample, which is linked to the decay in performance during cycles 10–20.^23^

Ex situ Bi-L3 XANES (Figure 4c) and FT EXAFS (Figure 4d) measured on the same samples clearly show that the oxidation state of Bi is reduced together with the amount of Bi–O bonds as a function of cycle number. LCF of the XANES curves by using Bi_2_O_3_ as Bi^3+^ reference and Bi metal foil as Bi^0^ reference estimated that the average oxidation state decreases from +1.9 after the first desodiation to +0.1 after the 10th desodiation (Figure 4e,f). At the same time, the characteristic signals from Bi metal at ∼3 Å are present in all the FT EXAFS plots (Figure 4d). These observations can also be linked to the increase in Bi particle sizes. When the Bi (nano)particles grow, the interface area toward the Na–Mo–O matrix decreases together with the number of Bi–O bonds.

To strengthen this hypothesis, we calculated the surface-to-volume ratio of theoretical cubic Bi particles with primitive packing and an atomic diameter of 3.3 Å, which is the average of the two closest Bi–Bi bonds in Bi metal (3.07 and 3.53 Å) (black curve in Figure 4f). By assuming that all the surface atoms are fully oxidized to Bi^3+^ and the rest of the atoms are Bi^0^ the average oxidation state of the particles will be given by the right axis in Figure 4f. Comparing this with the average oxidation state and crystallite sizes estimated from XANES and XRD, respectively, for the desodiated ex situ samples it is clear that this model fits very well for the first two cycles (red curve, Figure 4f). For the 4th, 5th and 10th cycle there is an increasing deviation between the calculated model and the estimates based on experimental data. Our assumption of perfectly cubic particles is probably not correct and will have larger influence for larger particles as they will tend to minimize the surface to volume ratio by forming more spherical particles. However, the main reason for the deviation is likely the deactivation of the electrochemical oxidation of Bi followed by deactivation of the Bi ⇋ NaBi reaction that has been shown to occur gradually during the first 10 cycles.^24^ Nevertheless, this analysis shows that our hypothesis of Bi–O bonds at the interface between the Bi particles and the Na–Mo–O matrix is reasonable.

### 3.5 Summary of Cycling and Degradation Mechanisms

**Figure 5:**
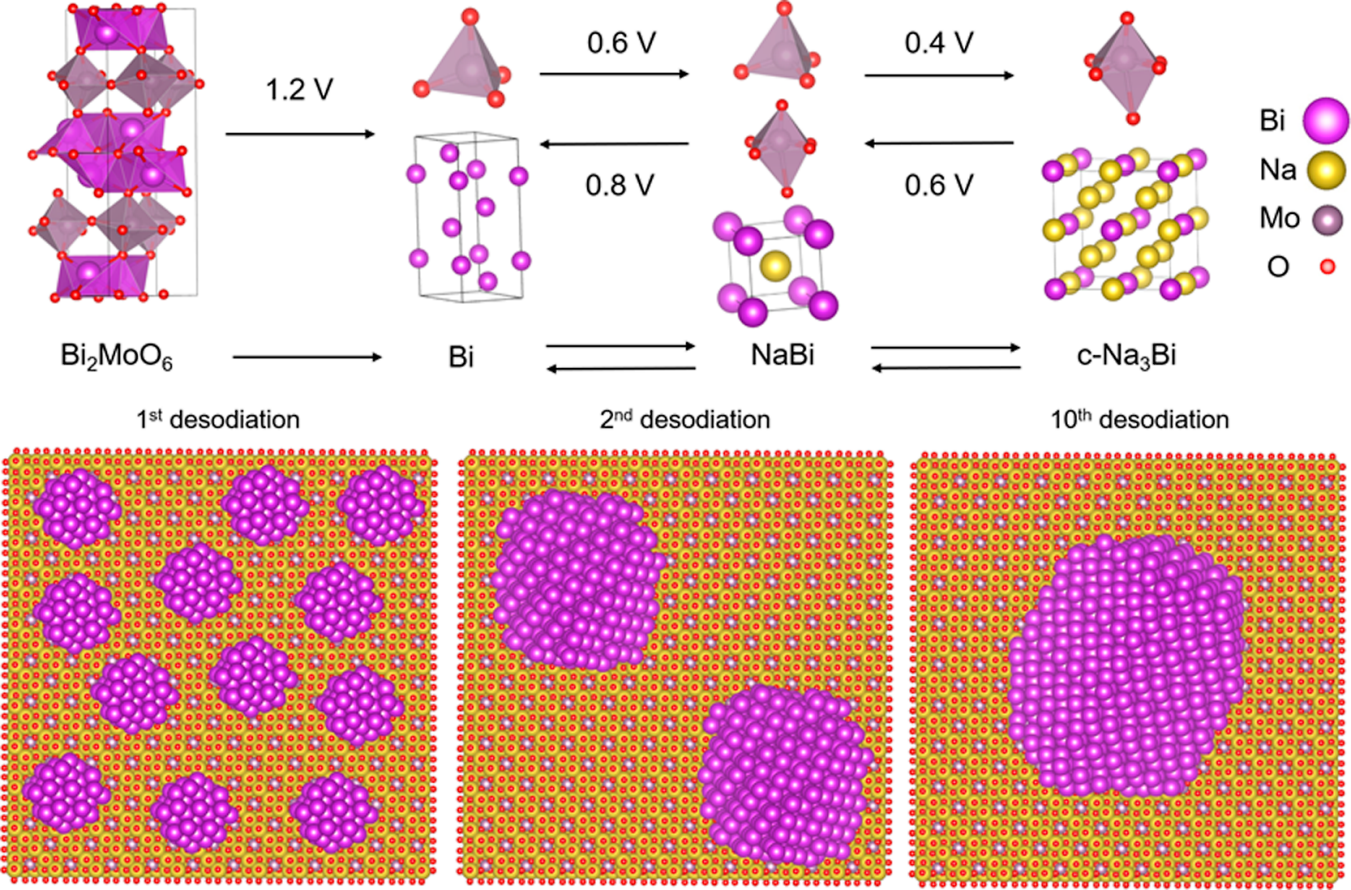
Graphical illustration of the cycling and degradation mechanism of Bi_2_MoO_6_. The top panel illustrates the cycling mechanism where Bi_2_MoO_6_ irreversibly converts into Bi (which further reversibly alloys to NaBi and Na_3_Bi) in a Na–Mo–O matrix with tetrahedrally coordinated Mo, which further shifts into distorted octahedral coordination. The bottom panel is a graphical illustration of the growth of the Bi crystallites with increasing cycle number.

The unique quasi-simultaneous operando PDF/XAS measurement (Sections 3.2 and 3.3) combined with extensive ex situ PDF and XAS measurements at different stages of cycling (Sections 3.4 and S7–S10, Supporting Information) provided a detailed description of the (de)sodiation mechanisms in Bi_2_MoO_6_ (Figure 5). During the first sodiation at ∼1.2 V vs Na/Na^+^ an irreversible conversion reaction occurs, in which Bi_2_MoO_6_ converts into Bi nanoparticles embedded in a Na–Mo–O matrix containing Mo^6+^ tetrahedrally coordinated to O^2–^ with a local atomic structure similar to that of Na_2_MoO_4_. The Bi particles alloy with Na forming NaBi at ∼0.6 V vs Na/Na^+^ and c-Na_3_Bi at ∼0.4 V vs Na/Na^+^. During the second step of the alloying reaction, the local coordination around Mo^6+^ in the Na–Mo–O matrix changes toward an octahedral arrangement, possibly similar to that in Na_4_MoO_5_ (Figure 5, top panel). During desodiation, c-Na_3_Bi transforms back to NaBi at ∼0.6 V and further to Bi at ∼0.8 V, while Mo^6+^ reverts to tetrahedral coordination. At the end of desodiation the surface atoms of the Bi particles bind to O in the Na–Mo–O matrix, leading to a positive average oxidation state for Bi. This oxidation provides additional capacity in the electrochemical measurements above 1 V vs Na/Na^+^ (Figure 1).

The average size of the Na_*x*_Bi particles increases as a function of cycle number due to coalescence of neighboring particles (schematically illustrated in Figure 5, bottom panel). This growth leads to a decrease in the surface-to-volume ratio of the Bi particles and fewer Bi–O bonds at the interface with the Na–Mo–O matrix. It also increases the distance between the alloying particles, reducing the ionic and electronic conduction and deactivating the electrochemistry. The apparent coalescence of the alloying particles is likely linked to the structural changes in the surrounding matrix, occurring at ∼0.7 V during desodiation, where the coordination of Mo^6+^ changes from distorted octahedral to tetrahedral as this could allow for the Na_*x*_Bi particles to move around, meet and coalesce. This structural change could be a consequence of the large volume work that occurs during the (de)alloying reactions (218% expansion from Bi → c-Na_3_Bi), which induces strain in the matrix. Avoiding these structural changes in the matrix by cycling between 0.01 and 0.70 V vs Na/Na^+^ instead of 0.01–2.00 V increased the cycling stability drastically. This result indicates that the structural changes in the matrix, resulting in coalescence of the alloying particles, were detrimental to the electrochemical performance.

## 4 Conclusion

The cycling and degradation mechanisms of Bi_2_MoO_6_ as an anode material for NIBs are complex. In this work, we have described previously unknown features of these mechanisms using quasi-simultaneous operando PDF and XAS (first 1.5 cycles) supported by ex situ measurements at different stages of cycling from the first 20 cycles. The results show that the Na–Mo–O matrix formed around Bi nanoparticles during the initial conversion reaction change local atomic structure during cycling, with the Mo^6+^ alternating between tetrahedral and distorted octahedral coordination. This structural change allows for movement and coalescence of the Na_*x*_Bi alloying particles. The combination of particle growth and increasing separation of the particles by growing amounts of poorly conducting matrix deactivate the electrochemistry between cycles 10–20. Reduction of the upper cutoff voltage to 0.70 V vs Na/Na^+^, which isolates the NaBi ⇋ Na_3_Bi reaction and prevents the structural change in the Na–Mo–O matrix, increases the cycling stability significantly during GC. This provides another indication for our hypothesis that a structurally stable matrix, which limits the coalescence of the alloying particles, is a key element in the recipe for designing CAMs with good cycling stabilities.

## Data Availability

The background data for this publication including procedures and scripts for data treatment are available at dataverse.no.^48^ The raw data files from the synchrotron experiments are stored at data.esrf.fr and will automatically be openly available in 2026.^50,51^

## Supporting Information Available

Ex situ XRD, PDF, XANES and EXAFS data on pristine and cycled Bi_2_MoO_6_ and reference materials; GC of Bi_2_MoO_6_; details on removal of Na and Cu peaks from XRD data before conversion to PDF; operando XRD and PDF data, including surface Rietveld refinement and non-negative matrix factorization analysis; description of experimental considerations regarding ex situ vs operando characterization (PDF)
