# Supplementary material for: (De)sodiation Mechanism of Bi2MoO6 in Na-Ion Batteries Probed by Quasi-Simultaneous Operando PDF and XAS
Source: Chem Mater. 2024 Aug 2;36(15):7514–24. doi: 10.1021/acs.chemmater.4c01503 (PMC11325532; doi:10.1021/acs.chemmater.4c01503)
Supplement: Supplementary file 1 — cm4c01503_si_001.pdf [file cm4c01503_si_001.pdf]

Supporting Information for:
"(De)sodiation Mechanism of Bi_2MoO_6 in Na-ion Batteries Probed by Quasi-Simultaneous *Operando* PDF and XAS"

Anders Brennhagen[1]*, Amalie Skurtveit[1], David S. Wragg[1,2], Carmen Cavallo[3], Anja O. Sjåstad[1], Alexey Y. Koposov[1,2], Helmer Fjellvåg[1]*

[1]Centre for Materials Science and Nanotechnology, Department of Chemistry, University of Oslo, PO Box 1033, Blindern, 0315, Oslo, Norway

[2]Department of Battery Technology, Institute for Energy Technology (IFE), Instituttveien 18, 2007, Kjeller, Norway

[3]FAAM, Strada Statale Via Appia 7 bis – 81030, Teverola (CE), Italy

E-mail: anders.brennhagen@smn.uio.no and helmer.fjellvag@kjemi.uio.no

Contents

Section S1 − Structural Characterization of Pristine Bi_2MoO_6 and References 2

Section S2 − Electrochemical Performance of Bi_2MoO_6 at Different Cycling Conditions 6

Section S3 − Removal of Na and Cu Peaks Before PDF Conversion 8

Section S4 − *Operando* XRD and PDF 9

Section S5 − Non-negative Matrix Factorization (NMF) 10

Section S6 − Note on Ex Situ vs *Operando* Characterization 12

Section S7 − Ex Situ XRD and PDF 14

Section S8 − Ex Situ XANES and EXAFS on Bi L3 Edge 19

Section S9 − Ex Situ XANES and EXAFS on Mo K Edge 23

Section S10 − Fitting of Sodiated Bi_2MoO_6 26

References: 27

Section S1 – Structural Characterization of Pristine Bi_2MoO_6 and References

X-ray absorption spectroscopy (XAS), X-ray diffraction (XRD) and pair distribution function (PDF) data were collected and analyzed for pristine Bi_2MoO_6 and all the reference materials used in this work (Figure S1−S3). The experimental data was matched with crystal structures obtained from literature, which are reported in Table S1.

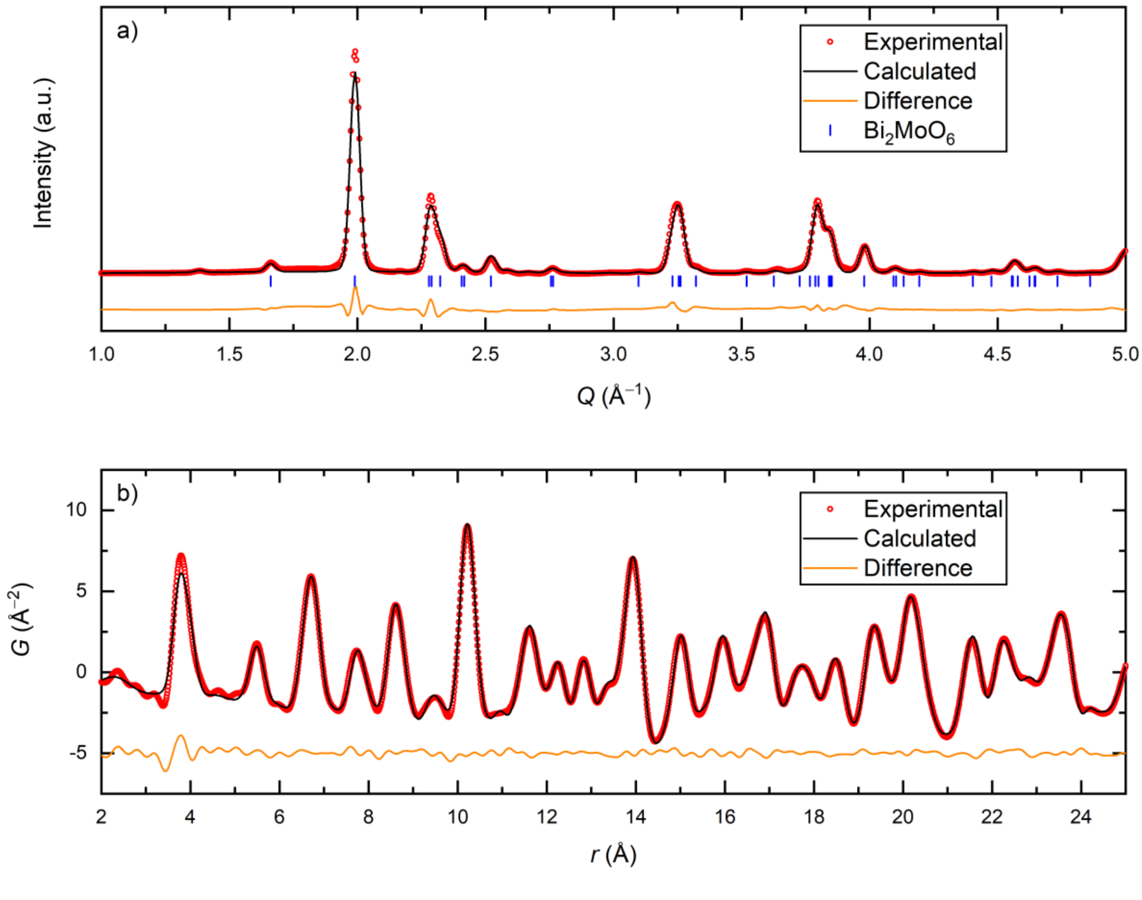

Figure S1: a) XRD pattern of pristine Bi_2MoO_6 mixed with 20 wt% Super P and b) corresponding PDF. Fitting of the data was performed in Topas v6.[1]

The X-ray absorption near edge spectroscopy (XANES) edge position of the Bi L3 edge in Bi_2MoO_6 overlaps with Bi_2O_3 indicating that both phases have +3 as the average oxidation state of Bi (Figure S2a). The edge position of Bi metal is at a significantly lower energy since the oxidation state is 0. The Fourier transformed (FT) extended X-ray absorption fine structure (EXAFS) data of Bi_2MoO_6 and Bi_2O_3 clearly show Bi−O bonds between 1−2 Å (Figure S2b). Between 2−3 Å they also show Bi−O bonds, but from oxygen atoms that are not in the closest coordination shell around Bi. In the region between 3.3−4.0 Å both materials show Bi−Bi interactions and Bi_2MoO_6 has in addition some overlapping Bi−Mo interactions. The peak for Bi_2MoO_6 in this region has a peak position of ~3.6 Å and corresponds to the peak observed in PDF around 3.8 Å. The *R* values presented in FT EXAFS are not directly real atomic distances, but related to them, and are often shifted slightly compared to the real *r* values that we obtain from PDF. To obtain real *r* values from EXAFS it is necessary to fit the curves where *r* is a fitting parameter. The closest Bi−Bi bonds in Bi metal are evident through peaks between 2.0−3.3 Å.

XANES measurements on the Mo K edge of Bi_2MoO_6 (Figure S2c) showed a pre-edge with low intensity resembling the MoO_3 reference that have distorted octahedral Mo−O coordination (close to 5-coordinated). This is in line with the results obtained from XRD where the structure of Bi_2MoO_6 (COD 1530868) have distorted Mo−O octahedra.[2] The edge position in the XANES spectra of Bi_2MoO_6 overlaps with both MoO_3 and Na_2MoO_4, indicating that the average oxidation state of Mo is +6. MoO_2 and Mo metal have edge positions at significantly lower energies due to their oxidation states of +4 and 0, respectively. Na_2MoO_4 has a significantly higher pre-edge peak compared to MoO_3 and Bi_2MoO_6 due to the tetrahedral coordination of Mo to O (Figure S2c). In the EXAFS FT spectrum (Figure S2d) we mostly observe Mo−O interactions, for Bi_2MoO_6, MoO_3, MoO_2 and Na_2MoO_4 between 1−2 Å.

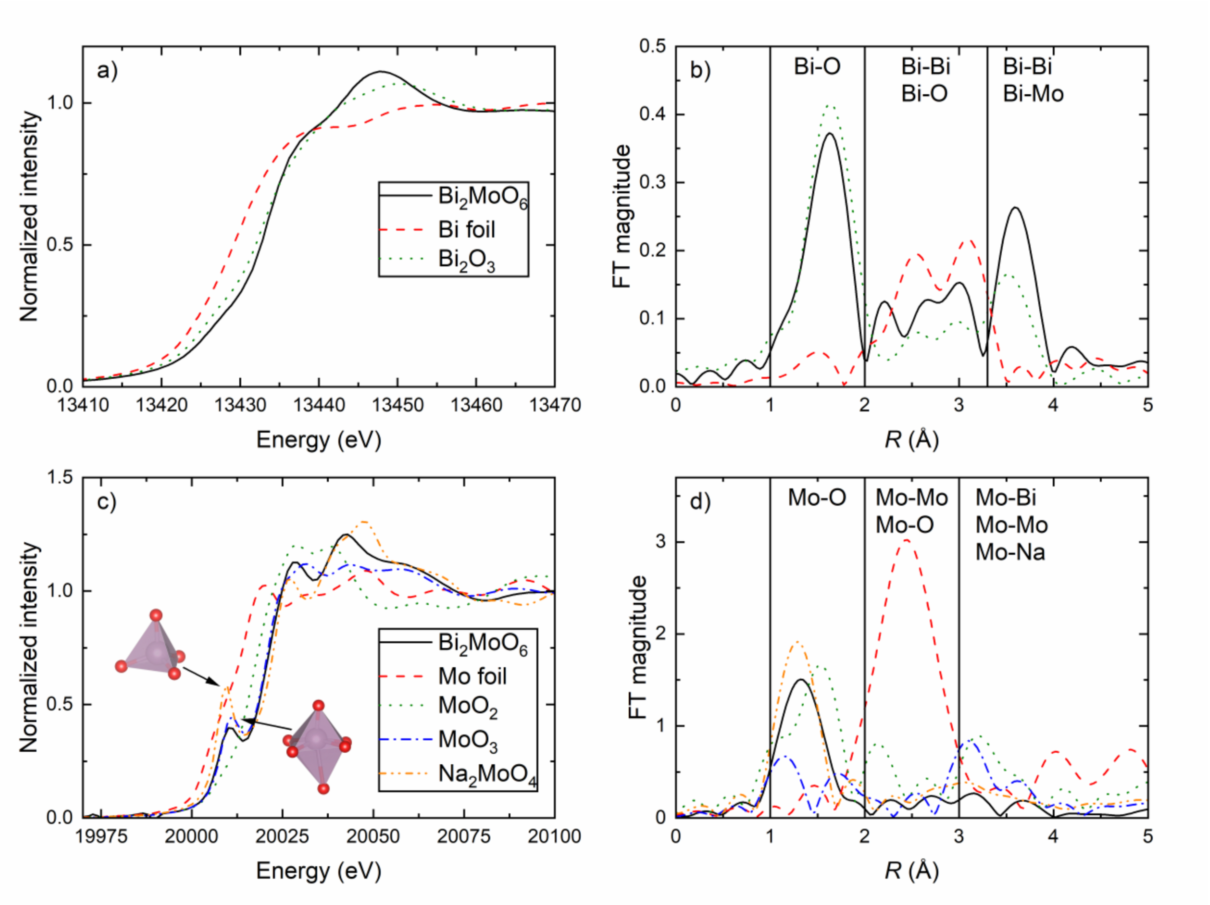

Figure S2: Bi_2MoO_6 compared to various Bi and Mo references. a) XANES Bi L3 edge, b) corresponding FT EXAFS graphs, c) XANES of Mo K edge and d) corresponding FT EXAFS graphs.

It is worth noticing that Bi_2MoO_6 with distorted octahedral coordination of Mo−O have a broader and less intense peak than Na_2MoO_4 (Figure S2d) The Mo−O peak in Na_2MoO_4 is also at lower *R* values than the Mo−O peak for Bi_2MoO_6. This is because the Mo−O bonds in tetrahedral coordination are shorter and more defined (less possibilities of distortion and varying lengths), while the Mo−O bonds in Bi_2MoO_6 are in general longer and have a larger distribution of distances. Between 2−3 Å, the closest Mo−Mo bonds from Mo metal and MoO_2 are present, while above 3 Å we have peaks corresponding to Mo−Mo distances for Bi_2MoO_6, Na_2MoO_4 and MoO_3. In addition, in this region, there are Mo−Bi distances for Bi_2MoO_6 and Na−Mo distances for Na_2MoO_4.

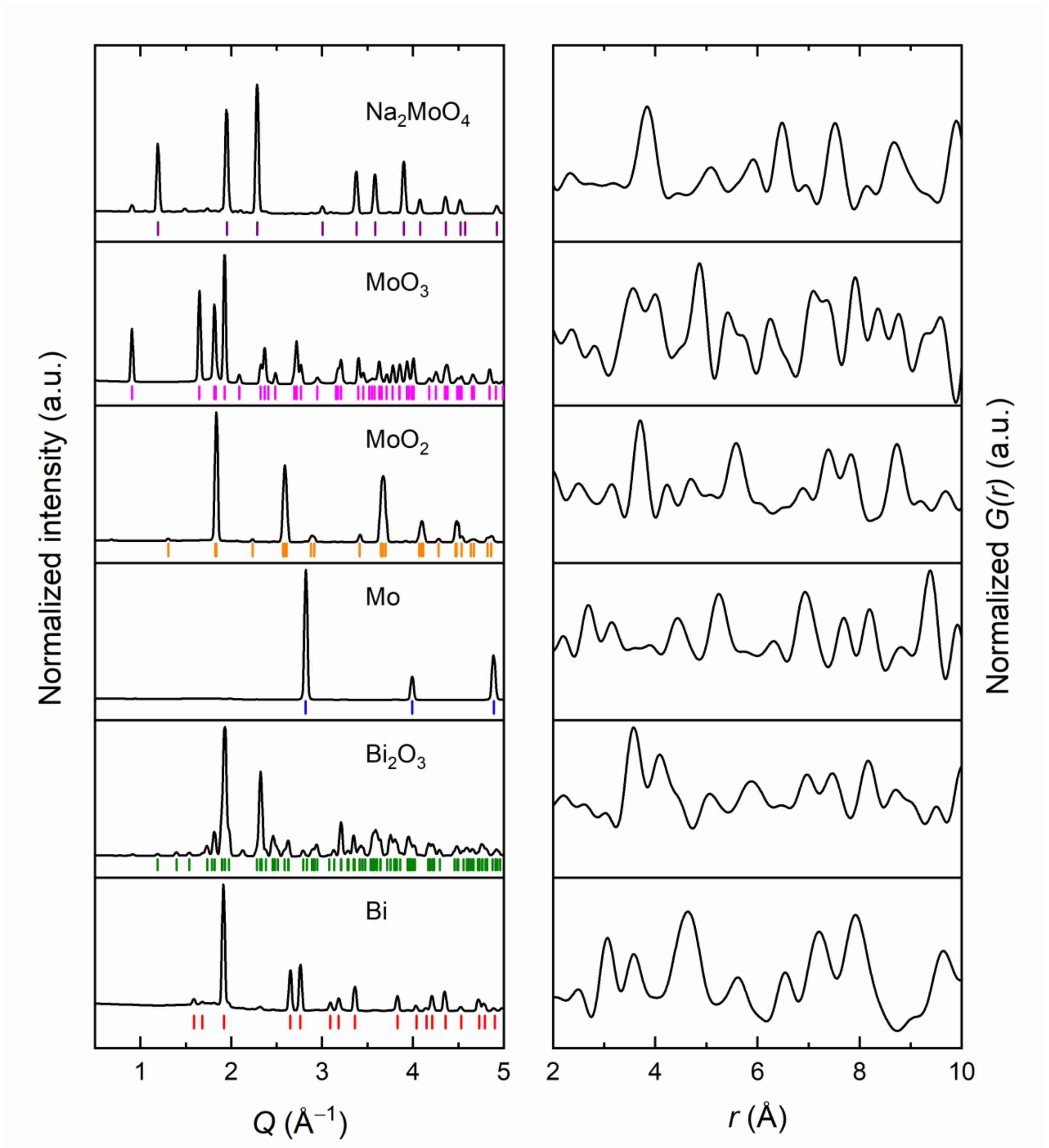

Figure S3: X-ray diffractograms (left) and PDFs (right) of reference materials measured at BM31 at ESRF. Phase information for corresponding crystal structures obtained from literature is provided in Table S1.

Table S1: Structural information of the phases used for XRD analysis, extracted from crystallographic information files (CIFs) obtained from crystallography open database (COD) and inorganic crystal structure database (ICSD).

Phase	Space group	Z	Unit cell parameters (Å)	Atomic sites					Source
				Atom	Wyckoff	x	y	z	
Bi_2MoO_6	$Pca2_1$ (29)	4	a = 5.4896	Bi1	4a	0.519	0.4231	0.983	COD:
			b = 16.22658	Bi2	4a	0.483	0.0786	0.989	1530868
			c = 5.5131	Mo1	4a	0.001	0.2479	0	
				O1	4a	0.049	0.1428	0.091	
				O2	4a	0.259	0.9956	0.271	
				O3	4a	0.242	0.5048	0.272	
				O4	4a	0.698	0.2297	0.251	
				O5	4a	0.207	0.2624	0.36	
				O6	4a	0.572	0.3602	0.575	
Bi	R-3m (166)	6	a = 4.546	Bi1	6c	0	0	0.23389	COD:
			c = 11.862						2310889
NaBi	P4/mmm	1	a = 3.46	Bi1	1a	0	0	0	ICSD:
	(123)		c = 4.8	Na1	1d	1/2	1/2	1/2	58816
h-Na_3Bi	$P6_3/mmc$	2	a = 5.448	Bi1	2c	1/3	2/3	1/4	COD:
	(194)		c = 9.655	Na1	2b	0	0	1/4	1010291
				Na2	4f	1/3	2/3	0.583	
c-Na_3Bi	Fm-3m (225)	4	a = 7.66526	Bi1	4a	0	0	0	[3]
				Na1	4b	1/2	1/2	1/2	
				Na2	8c	1/4	1/4	1/4	
Bi_2O_3	$P2_1/c$ (14)	4	a = 5.8486	Bi1	4e	0.524	0.1831	0.3613	COD:
			b = 8.1661	Bi2	4e	0.0409	0.0425	0.7762	9012546
			c = 7.5097	O1	4e	0.780	0.3	0.0114	
			β = 113	O2	4e	0.242	0.044	0.0152	
				O3	4e	0.271	0.024	0.01013	
Mo	Im-3m (229)	2	a = 3.1473	Mo1	2a	0	0	0	COD:
									9008543
Na_2MoO_4	Fd-3m (227)	8	a = 9.10985	Na1	16c	0	0	0	ICSD:
				Mo1	8b	0.375	0.375	0.375	14555
				O1	32e	0.26172	0.26172	0.26172	
MoO_3	Pbnm (62)	4	a = 3.9621	Mo1	4c	0.075	0.102	1/4	COD:
			b = 13.855	O1	4c	0.526	0.426	1/4	9009670
			c = 3.6986	O2	4c	0.566	0.086	1/4	
				O3	4c	0.01	0.224	1/4	
MoO_2	$P2_1/c$ (14)	4	a = 5.6102	Mo1	4e	0.2309	-0.0088	0.0147	COD:
			b = 4.8573	O1	4e	0.1138	0.2212	0.2345	1548687
			c = 5.6265	O2	4e	0.3901	0.6981	0.2985	
			β = 120.915						

Table S2: Close Bi–Bi and Bi–Na bond distances given in Å calculated from the CIF files for the reference phases presented in Table S1. Due to the large number of Bi–Bi distances in Bi_2O_3 these are grouped together where the number in parenthesis indicate how many discrete distances the structure has within the ranges.

Coordination shell	Bi_2O_3	Bi	NaBi	c-Na_3Bi	h-Na_3Bi
1	3.46–3.72 (5)	3.07 (Bi–Bi)	3.43 (Bi–Na)	3.32 (Bi–Na)	3.15 (Bi–Na)
2	3.91–4.13 (5)	3.53 (Bi–Bi)	3.46 (Bi–Bi)	3.83 (Bi–Na)	3.22 (Bi–Na)
3	4.27–4.38 (3)	4.55 (Bi–Bi)	4.80 (Bi–Bi)	5.42 (Bi–Bi)	3.54 (Bi–Na)
4		4.75 (Bi–Bi)	4.89 (Bi–Bi)		5.45 (Bi–Bi)

Section S2 – Electrochemical Performance of Bi_2MoO_6 at Different Cycling Conditions

The electrochemical performance of Bi_2MoO_6 was evaluated through galvanostatic cycling (GC) at different current densities and voltages ranges (Figure S4 and Figure S5). Cycling between 0.01−2.00 V vs Na/Na^+ with current densities in the range of 0.02−1.00 A g^{-1} showed only small variations in performance (Figure S4). The most pronounced difference was the increased overpotential in the (de)sodiation curves when current densities were elevated.

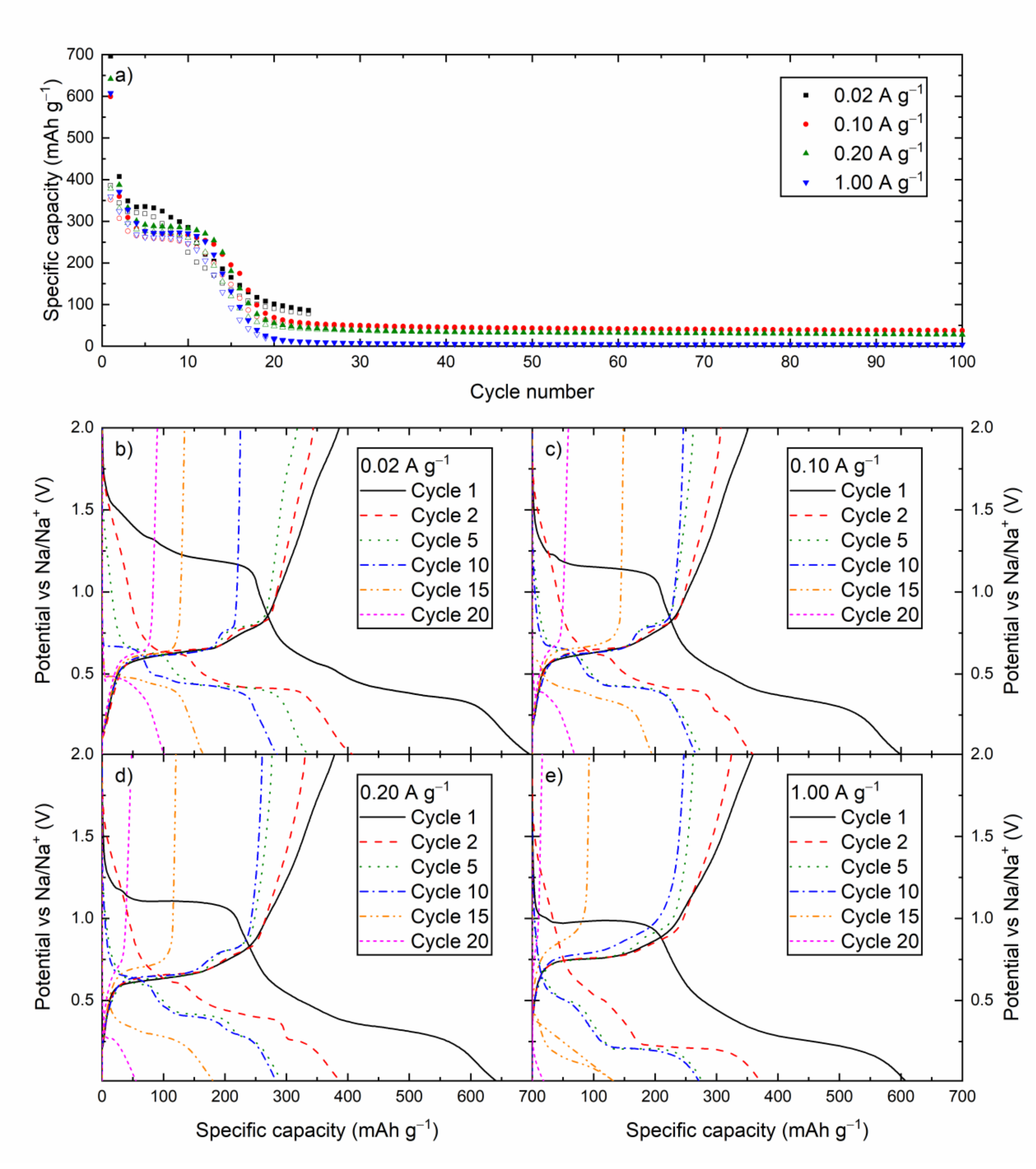

Figure S4: GC measurements of Bi_2MoO_6 cycled between 0.01−2.00 V vs Na/Na^+ at different current densities. a) Specific capacity as function of cycle number where closed symbols represent sodiation capacities and open symbols represent desodiation, b)-e) selected (de)sodiation curves from the corresponding measurements. Current densities are specified in the legends.

By avoiding the NaBi−Na_3Bi reaction, through increasing the lower cut-off voltage to 0.50 V vs Na/Na^+ during cycling at 0.1 A g^{-1}, the cycling stability increased slightly. However, the capacity was consistently lower than during cycling between 0.01−2.00 V (Figure S5a and d). Reducing the upper cut-off voltage to 0.70 V, thus avoiding the Bi−NaBi reaction and isolating the NaBi−Na_3Bi reaction, had a drastic impact on the cycling stability. Even if the reduced cut-off voltage limited the capacity to ~200 mA g^{-1} the stability increased enough to outperform the cycling between 0.01−2.00 V after 20 cycles (Figure S5a−b). The isolated NaBi−Na_3Bi reaction could also handle cycling at 1 A g^{-1} and maintain the capacity even better than the measurement at 0.1 A g^{-1} (Figure S5a and c). In this high-rate measurement, it was necessary to increase the upper cut-off voltage to 0.80 V in order to account for the increased overpotential as a result of the higher current. The cycling performance of this material with the limited voltage window is still not good enough for commercial interest, but it is a significant improvement. More importantly these measurements show that it is mainly the Bi−NaBi reaction and the extra oxidation of Bi (discussed in Section S7−S8 and Section 3.4, main article) that are the most detrimental for the cycling stability of Bi_2MoO_6. This could also explain why these are the reactions that becomes inactive first during cycling for both Bi_2MoO_6,[2] and $BiFeO_3$.[4]

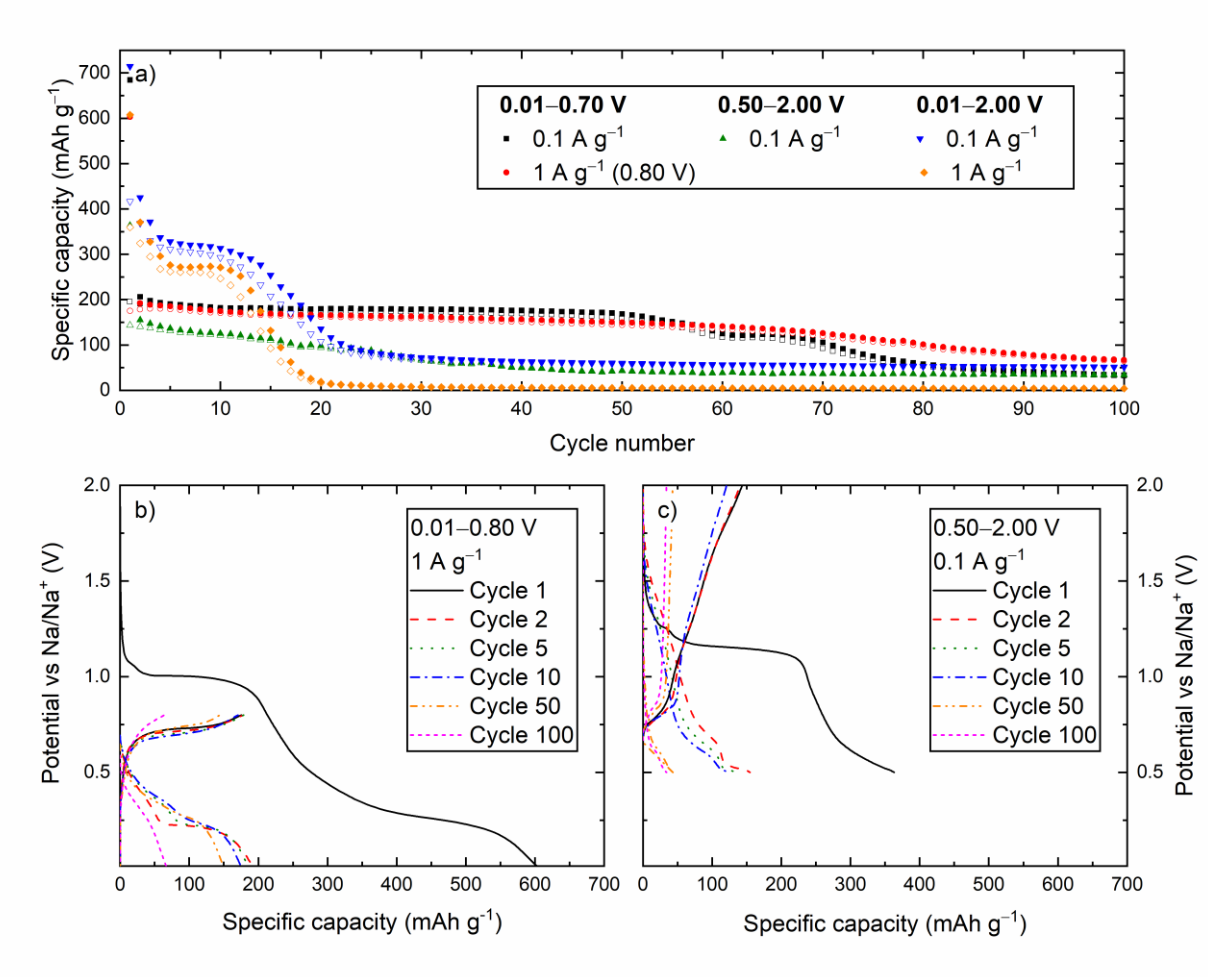

Figure S5: GC measurements of Bi_2MoO_6 at different current densities and voltage ranges. a) Capacity per cycle plot comparing the performance from the different measurements where closed symbols represent sodiation capacities and open symbols represent desodiation, b)-e) selected (de)sodiation curves from the corresponding measurements. Cycling conditions are specified in the legends.

Section S3 – Removal of Na and Cu Peaks Before PDF Conversion

In the collected *operando* XRD data, there were strong Bragg reflections of Cu from the current collector and Na from the counter electrode. Because of their high intensities, these signals needed to be removed before converting the diffractograms into PDFs. The Cu and Na peaks were subtracted by using Python version 3.11. The Python script (named "peakremoval_xy.py")[5] takes in the start and end positions of the peaks and replaces them with a straight line (Figure S6). This method allowed us to obtain decent PDF data, where the main features are trustworthy. However, it is by no means perfect, and small features might not represent the real system. When removing the Na and Cu peaks, the background in that area is turned into a straight line. In some cases, the Na and Cu peaks might also overlap with signals from the active material. This means that we do not only remove the unwanted peaks, but we also risk losing meaningful information. To obtain the best possible compromise we removed as little as possible, but as much as necessary by searching through all of the unwanted peaks in our XRD data and manually chose the start and end positions of each peak. Cutting away too little of the peaks led to peak residues that could, in the worst case, dominate the PDFs. Several of the Bi_2MoO_6 peaks partially overlapped with Na and Cu peaks (for example at ~2 Å in Figure S6a). In addition, the Bi_2MoO_6 peaks have much stronger intensities than the signals from the nanocrystalline Na_xBi phases. Therefore, we removed less from the peaks in the scans containing Bi_2MoO_6 (scan 0−4, Figure S6a) than we did for the rest of the scans (scan 5−29, Figure S6b). In this way, most of the Bi_2MoO_6 signals were maintained, while it was possible to observe weaker features from the alloying particles.

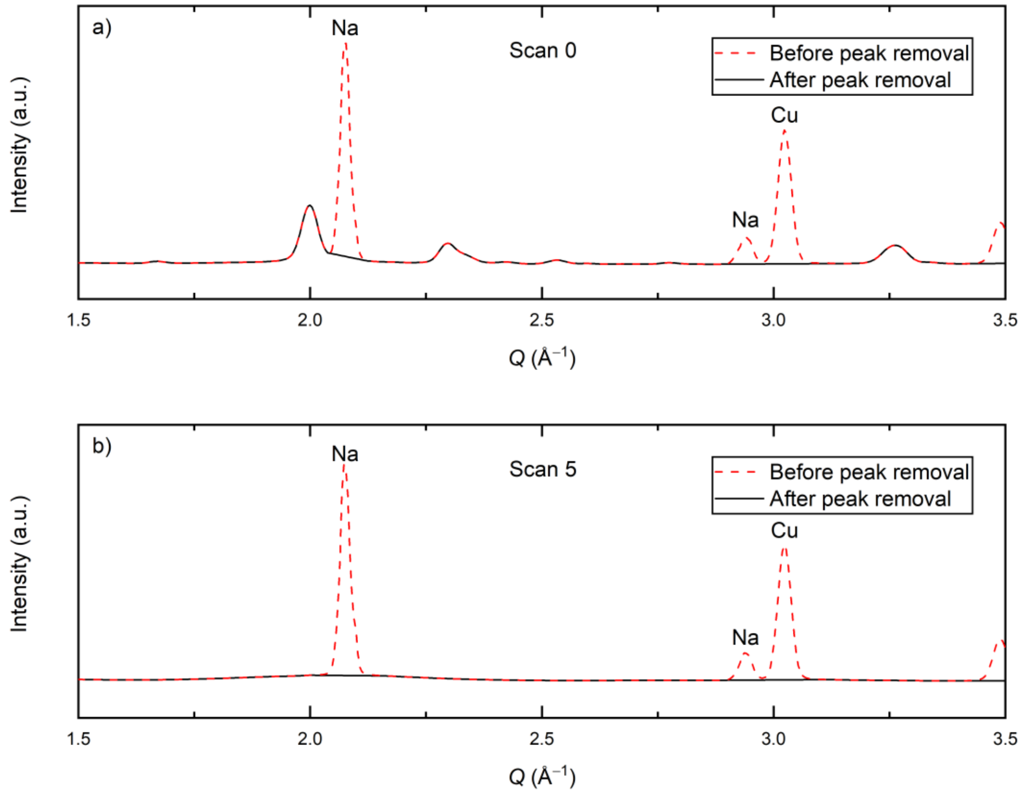

Figure S6: Example of removal of Na and Cu peaks from the XRD data before converting to PDF. a) Shows the removal of peaks from scan 0 and b) removal of peaks from scan 5.

Section S4 – *Operando* XRD and PDF

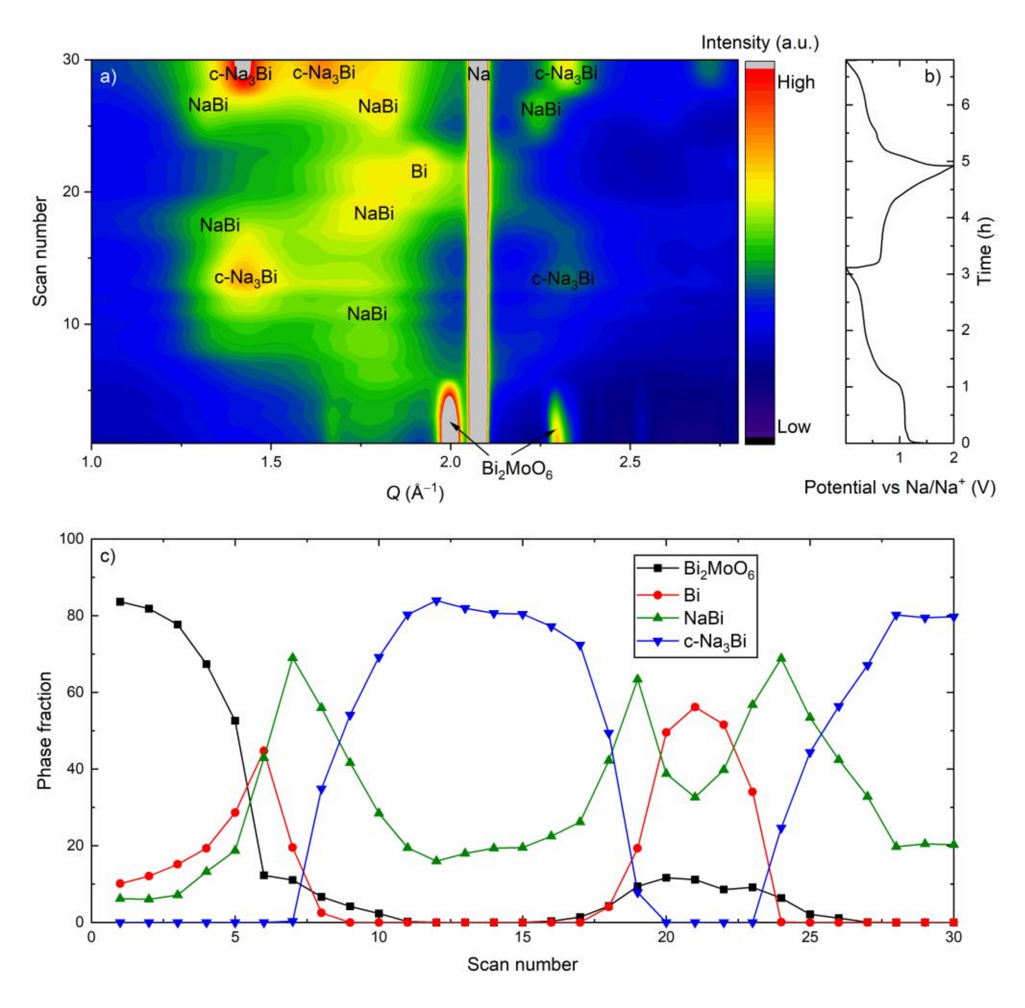

Figure S7: ***Operando*** **XRD of Bi_2MoO_6, extracted from the same measurement as Figure 2−3 in the main article. a) Contour plot, b) electrochemistry and c) phase fractions from surface Rietveld refinements. The refinements are performed on the data set after background subtraction and removal of Cu and Na peaks (i.e. the data set used for the conversion to PDF). The apparent emergence of Bi_2MoO_6 at first desodiation is probably not realistic but could be an indication of oxidized Bi.**

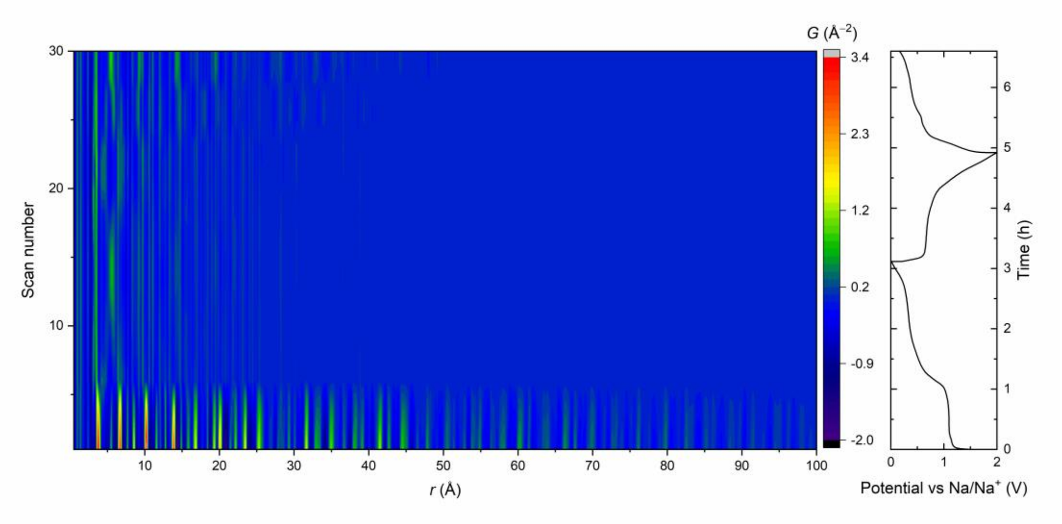

Figure S8: ***Operando*** **PDF over an *r* range of 0.5−100 Å, showing loss of long-range order after initial conversion reaction.**

Section S5 – Non-negative Matrix Factorization (NMF)

Obtaining reliable PDF data is challenging, especially when collected in *operando* mode, because of the various processing steps including subtraction of background signals. The dominant background signals in our *operando* measurement came from glassy carbon windows in the *operando* cells, Cu in the current collector and Na from the counter electrode. The removal of these signals and other processing steps may lead to artificial noise in the PDF data (Section S3). Therefore, it is important to critically assess the validity of the data and find support in other techniques, before trusting the results. As a part of evaluating and analyzing the *operando* PDF data, we performed non-negative matrix factorization (NMF) by utilizing "PDF in the cloud" in order to see if the mathematically extracted components fitted the expected chemical phases (Figure S9).[6, 7] Based on the *operando* XRD data, four main phases were expected: Bi_2MoO_6, Bi, NaBi and c-Na_3Bi (Figure S7). Therefore, we set the number of components to four in the NMF calculation. The four extracted components from NMF, did not fit perfectly with the theoretical phases, but the main features were the same (Figure S9a).

Phase 1 (Bi_2MoO_6) provided a good fit, as expected, due to the crystallinity of the pristine sample providing strong signals compared to the other phases (Figure S9a). Phase 2 (Bi) clearly showed signals for the two closest Bi−Bi distances at ~3 Å and ~3.5 Å, but the ratio of their intensities did not fit well with the theoretical Bi-metal phase (Figure S9a). This could be explained by the influence of Bi−O bonds leading to more Bi−Bi distances at ~3.5 Å, as discussed in Section 3.3 in the main article. Phase 3 (NaBi) showed most of the expected peaks with only small deviations in intensities and peak positions (Figure S9a). The main peaks for the c-Na_3Bi phase are expected to be at 3.32 Å, 3.83 Å and 5.42 Å (Table S2). In phase 4, we observe clear peaks at ~3.3 Å and ~5.4 Å that fits well with two of the expected values (Figure S9a). However, the Na−Bi bonds at 3.83 Å are not visible, and they are not observed in the contour plot from *operando* PDF either (Figure 3e, main article). This could be an indication of Na deficiency in the c-Na_3Bi phase, where the Na site that is needed for the Na−Bi bonds at 3.83 Å is not filled.

The deviations between the NMF components and the fits are likely due to two main effects: noise in the PDF data and disordered nanocrystalline/amorphous phases. The removal of Na and Cu peaks before the PDF conversion may have led to loss of information and introduction of non-real signals (Section S3). In addition, there are amorphous electrode components (Super P and binder) that were not subtracted from the dataset, and the Na−Mo−O matrix is not accounted for in the analysis. All of these factors contribute to the PDF signals and could explain some of the minor deviations in the data set. The other main effect is that the phases, except for Bi_2MoO_6, are nanocrystalline or amorphous and deviate from the perfect crystal structures. The local structure could be significantly different from the theoretical phases and better models are needed to obtain a good fit. The already mentioned effects of the Bi−O interactions and the possible Na deficiency are examples of local structural deviations that could partially explain the poor fits.

The evolution of the phase fractions (described from the NMF components) showed the same main trend as the surface Rietveld refinement from the *operando* XRD (Figure S9b, Figure S7c). This indicates that the extracted components describe the real chemical phases in the system

reasonably well and clearly show both the irreversible conversion reaction and the two-step alloying reaction. Hence, the main features of the PDF data are trustworthy.

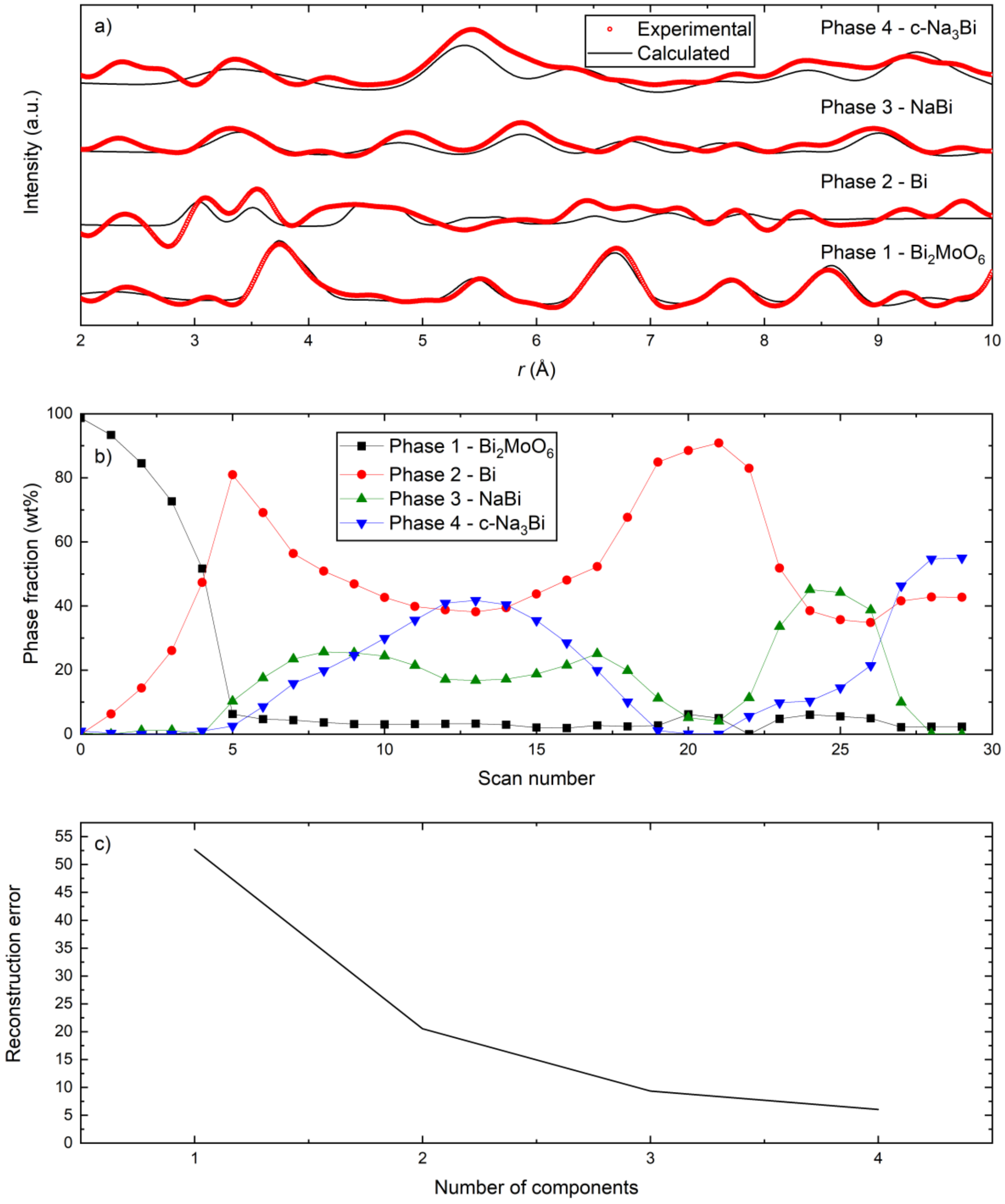

Figure S9: NMF results with four components from *operando* PDF measurement. a) Extracted components including fits of the expected chemical phases, b) phase fraction of the components as a function of scan number and c) reconstruction error as a function of number of components.

Section S6 – Note on Ex Situ vs *Operando* Characterization

One of the main advantages with *operando* characterization compared to ex situ is that the changes in the sample are measured in real time. This provides a greater confidence that the measured data is realistic and makes it possible to detect metastable phases. With ex situ samples, extracted from batteries, the sample could change or react before the measurement, which could provide misleading information regarding the actual chemical reactions occurring inside the battery. On the other hand, ex situ measurements in general provide better data quality and with some techniques, where *operando* measurements are not feasible, the only choice is ex situ. The combination of *operando* and ex situ measurements provides greater confidence in the results than each of the individual measurements, as long as they are coherent.

Throughout this work, we measured XRD/PDF and XAS on many ex situ capillaries in addition to the combined *operando* measurements. Several of these capillaries have shown unexpected results, as they probably reacted at some point between coin cell disassembly and the X-ray measurement. This is especially pronounced for sodiated samples (containing NaBi and Na_3Bi) as they are unstable in air. The ex situ samples are packed in capillaries and sealed in inert atmosphere and should in theory not be exposed to air or moisture, but apparently several of them had small leaks. The clearest examples are the ex situ samples cycled to 0.50 V during the 1st and 2nd sodiation (Figure S10). Both of these samples were expected to contain mainly NaBi, but they showed only diffraction peaks of Bi and Na_2MoO_4. In the XANES data, both samples show Bi L3 edge positions at significantly higher energies than the Bi-metal reference, proving that the oxidation state is positive rather than negative as it should have been in NaBi (Figure S10a). The oxidation state seems to be higher than most of the fully desodiated samples (Section S8) and higher than the scan measured at 2.00 V from the *operando* measurement (Figure 2, main article). Diffraction peaks of Na_2MoO_4 are not observed to any significant degree in any of the capillary measurements presented in Section S7. This could mean that the presence of crystalline Na_2MoO_4 indicates that the sample has reacted and the data is no longer trustworthy, which is a good way to separate reacted from unreacted desodiated samples where we do expect to have Bi metal.

Some of the measured samples showed the expected results during the XAS measurements, but had clearly reacted before the XRD measurements, which were carried out a couple of days later. One example is the sample extracted at 0.70 V from the 2nd desodiation, where the edge position of Bi indicates an oxidation state of slightly less than 0 (Figure S10a). This is close to what we expected and significantly different from the two reacted samples at 0.50 V. However, the XRD patterns of all 3 samples are close to identical showing both clear Bi and Na_2MoO_4 peaks, indicating that the 0.70 V sample had changed significantly between the XAS and the XRD measurement (Figure S10c).

These results show that there are some uncertainties with regards to the validity of ex situ samples in general and specifically for the data set presented in this article. The ex situ data presented in the later sections and in the main article are selected as the most reliable replicas from the data set. However, some of them might still have reacted slightly.

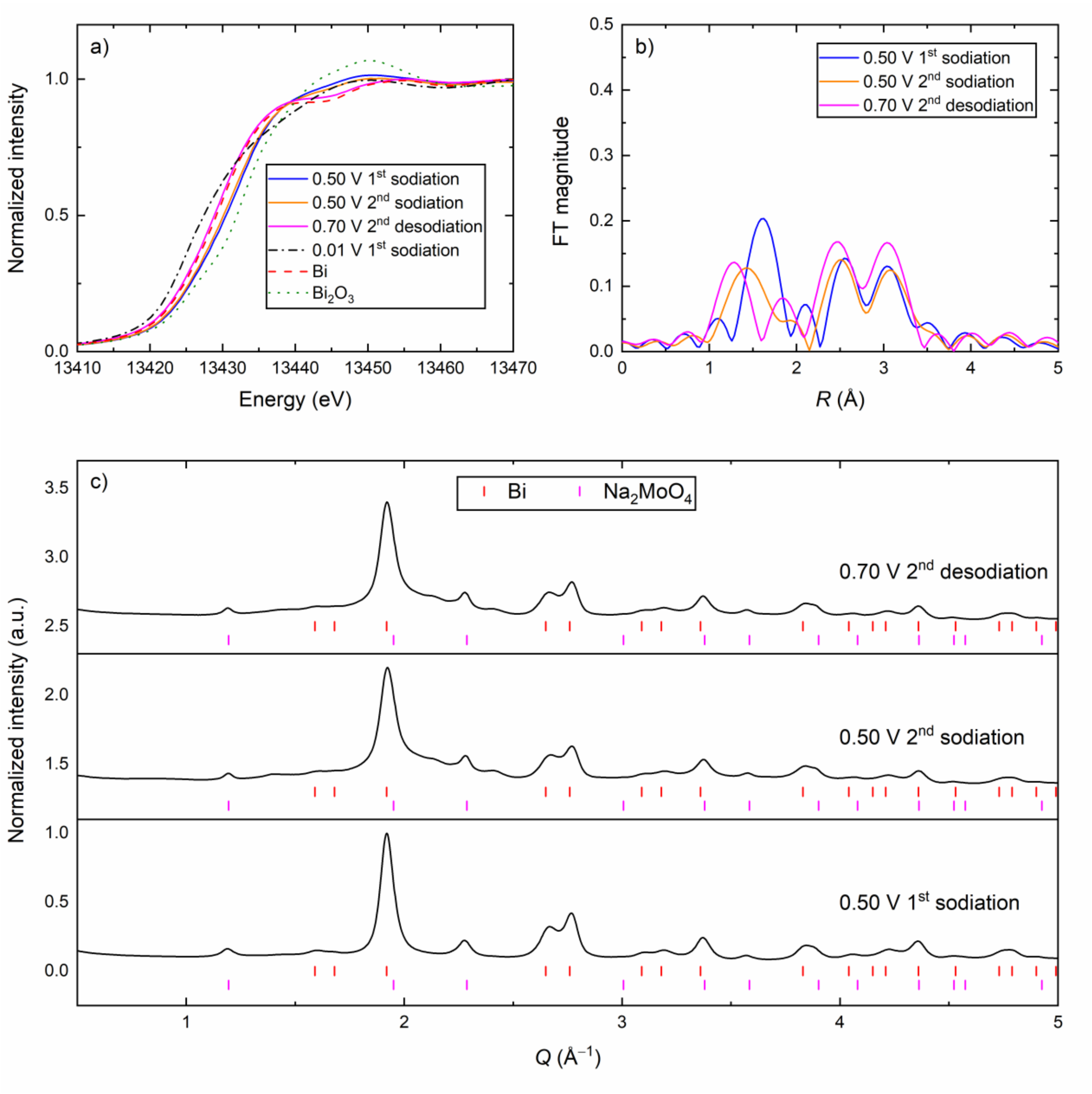

Figure S10: Examples of ex situ samples that reacted before or between measurements. a) Bi L3 XANES data vs references, b) FT EXAFS graphs, c) XRD data.

Section S7 – Ex Situ XRD and PDF

Figure S11 shows the XRD patterns and PDFs of Bi_2MoO_6 ex situ samples extracted at selected potentials during the first (de)sodiation cycle. Pristine Bi_2MoO_6 quickly becomes amorphous and seems to form something similar to Bi metal at 0.80 V. While at the fully sodiated state (0.01 V) mainly nanocrystalline c-Na_3Bi is present. During the first desodiation, the c-Na_3Bi particles transform back to metallic Bi. At 0.70 V we would expect to have NaBi, as observed for the 0.70 V sample from the 5th desodiation (Figure S13). However, only peaks corresponding to Bi are visible. This might mean that the sample have reacted, as discussed in Section S6. After full desodiation, at 2 V, the Bi peaks are significantly broader than for the samples extracted at 0.70 and 1.00 V (Figure S11). This is likely due to the Bi−O interactions forming between 1.00−2.00 V, as shown by XAS (Section S8), leading to a more distorted crystal structure. During the second sodiation, we observe a reversed reaction compared to the first desodiation where c-Na_3Bi is again formed (Figure S12). The diffraction peaks for both the Bi and the c-Na_3Bi phases are sharper during the 2nd cycle than the 1st, indicating growth in crystallite sizes. The ex situ samples from the 5th cycle also show the same trend where the peaks are even sharper (Figure S13). Here the oxidation of Bi metal is not that pronounced, and the Bi peaks are similar for the 0.80, 1.00 and 2.00 V samples. We were also a bit luckier with the 0.50 and 0.70 V samples as these show NaBi, as expected.

There are some clear trends when studying the fully sodiated (Figure S14) and desodiated samples (Figure S15) from different cycles. In the sodiated samples the diffraction peaks of c-Na_3Bi becomes sharper and sharper until the 10th sodiation where some h-Na_3Bi appear. This is consistent with our previous *operando* XRD study [2]. The sample from the 20th sodiation has probably reacted, as we would expect it to be a combination of c-Na_3Bi and h-Na_3Bi and not containing NaBi and Bi. All the fully desodiated samples (Figure S15) exhibit XRD peaks corresponding to Bi metal, except for the 20th desodiation where the peaks indicate some NaBi in addition. From our previous *operando* XRD measurement we would expect the sample to only consist of NaBi and maybe some Na_3Bi [2], so it might be that this capillary also have reacted a bit. Nevertheless, the presence of NaBi shows that the system is not able to fully desodiate to Bi after 20 cycles and is partially stuck in the sodiated state. The diffraction peaks from Bi in the desodiated samples become sharper with cycle number, indicating crystallite growth. The shoulder on the right side of the main Bi peak is also related to the oxidation of Bi between 1.00 and 2.00 V. This oxidation is reduced with cycle number (as discussed in Section S8 and Section 3.4, main article) together with the peak-shoulder. There are also clear changes in the PDFs for the desodiated sample as the ratio of the intensity of the peaks at ∼3 Å and ∼3.5 Å is changing. The peak at ∼3 Å corresponds to the closest Bi−Bi bonds in Bi metal, which are theoretically at 3.07 Å. The closest Bi−Bi distances in Bi_2O_3 are between 3.46−3.72 Å (Table S2), which corresponds quite well to the observed peak at ∼3.5 Å. This explains why increased oxidation of Bi leads to an increase in the intensity of this peak. The second Bi−Bi shell is theoretically at 3.53 Å and will therefore also contribute to the peak at ∼3.5 Å. This is why this peak is still present even when we do not have any significant oxidation of Bi.

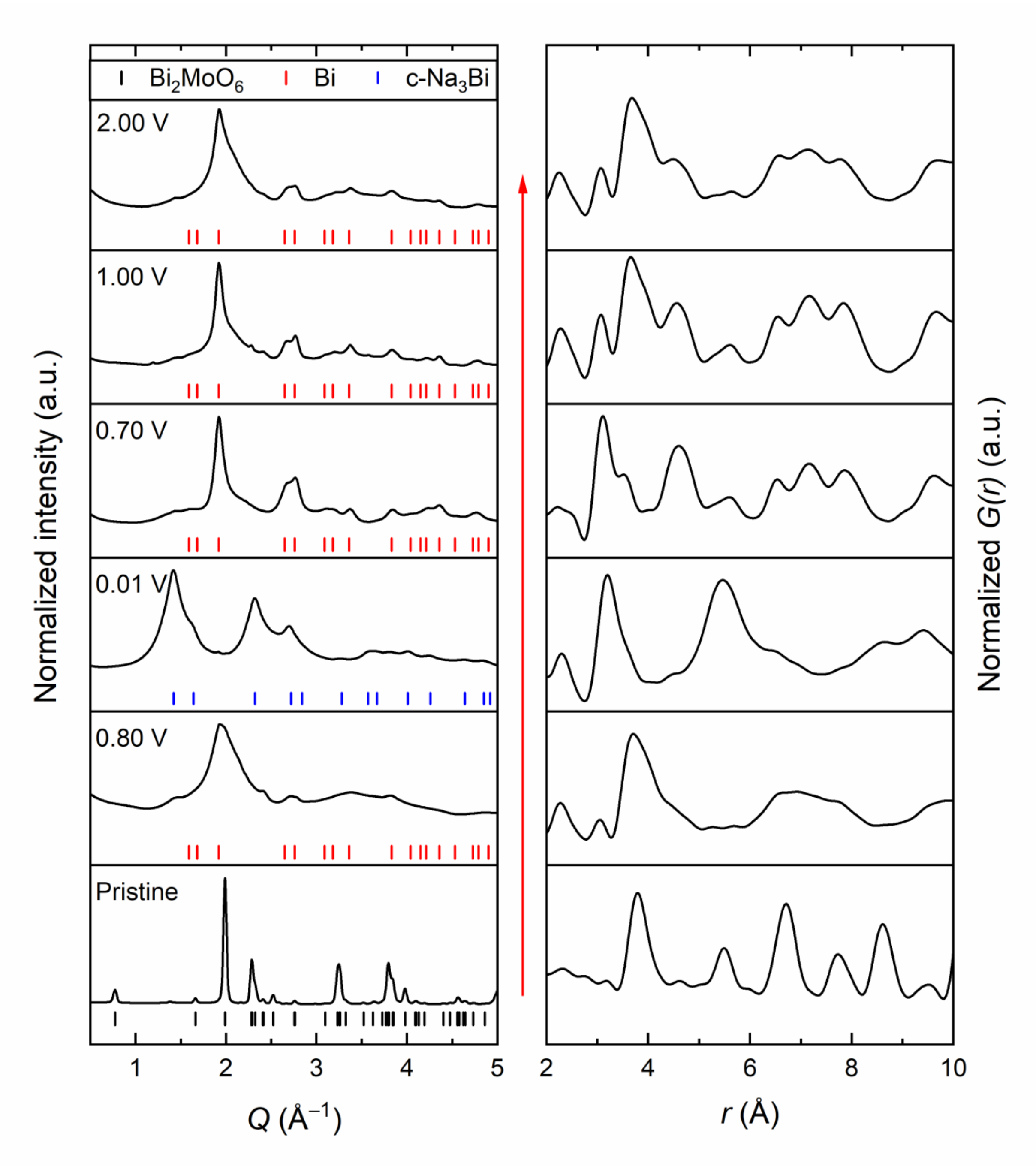

Figure S11: X-ray diffractograms (left) and corresponding PDFs (right) from ex situ measurements on Bi_2MoO_6 cycled to specific potentials vs Na/Na^+ during the 1st cycle. The red arrow indicates the direction of cycling.

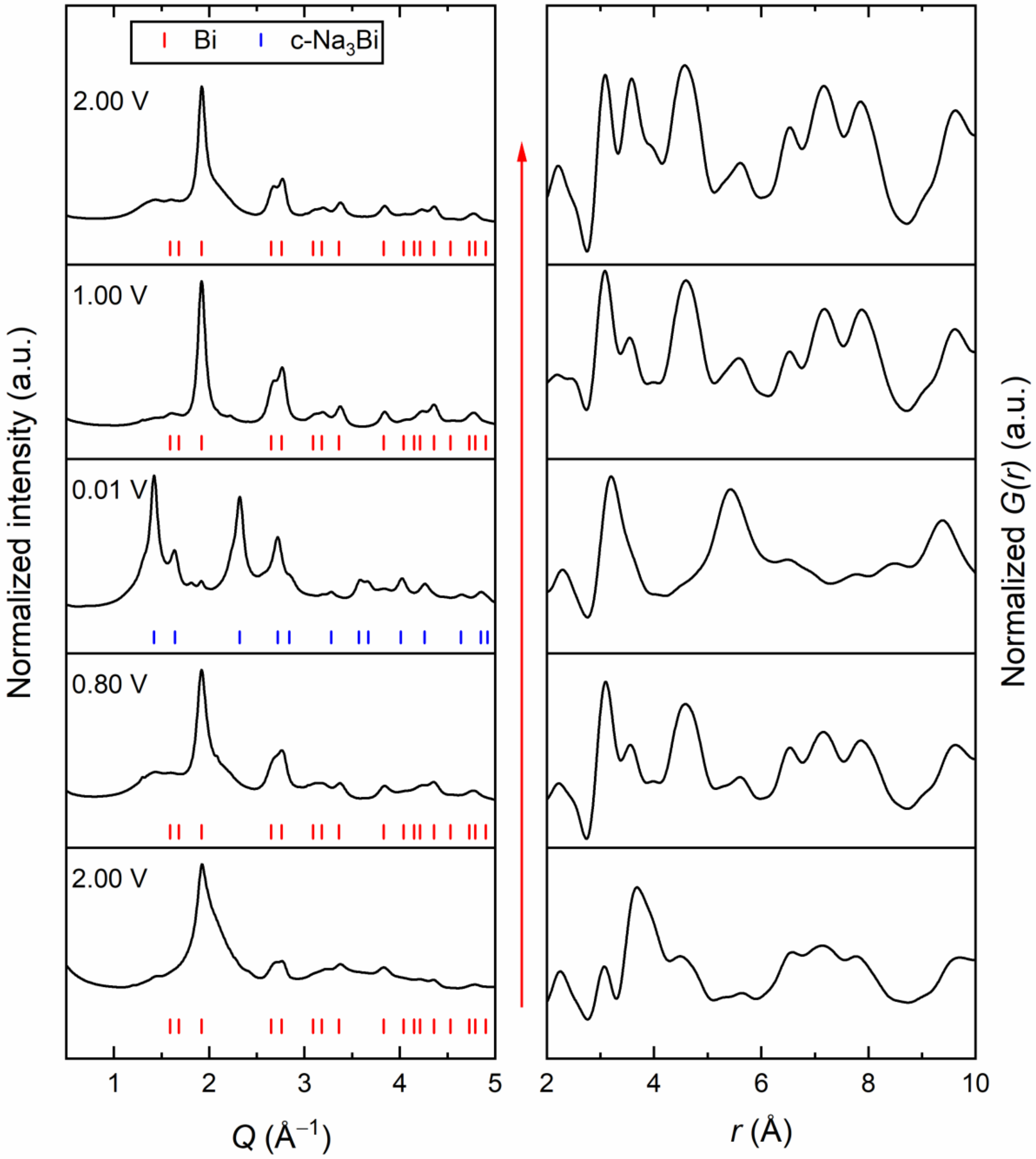

Figure S12: X-ray diffractograms (left) and corresponding PDFs (right) from ex situ measurements on Bi_2MoO_6 cycled to specific potentials vs Na/Na^+ during the 2nd cycle. The red arrow indicates the direction of cycling.

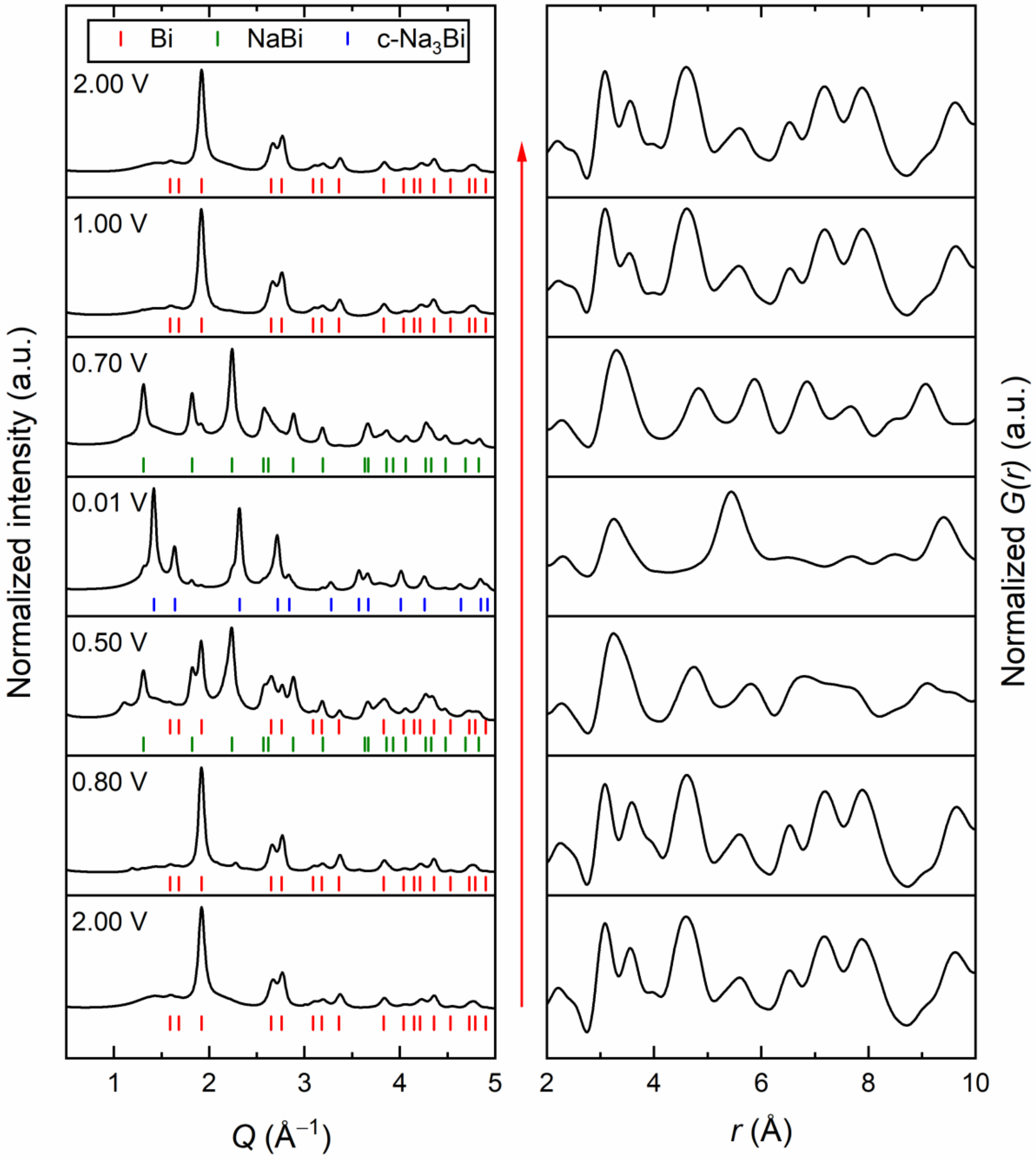

Figure S13: X-ray diffractograms (left) and corresponding PDFs (right) from ex situ measurements on Bi_2MoO_6 cycled to specific potentials vs Na/Na^+ during the 5th cycle. The red arrow indicates the direction of cycling.

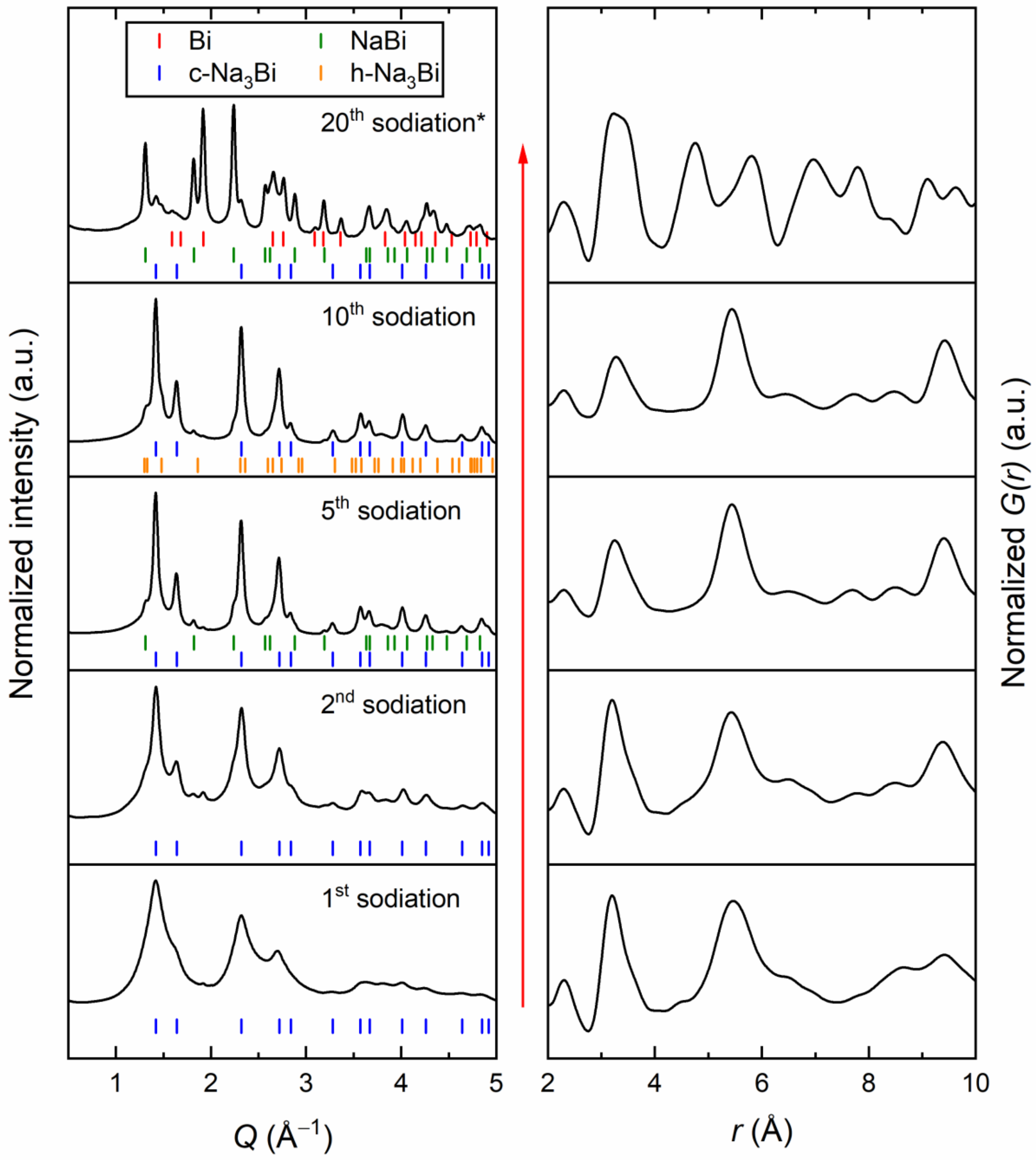

Figure S14: X-ray diffractograms (left) and corresponding PDFs (right) from ex situ measurements on Bi_2MoO_6 samples sodiated to 0.01 V vs Na/Na^+ from different cycles. The red arrow indicates the direction of cycling. *The sodiated sample from the 20th sodiation have probably reacted between coin cell disassembly and the X-ray measurements, as indicated by the significant amount of metallic Bi in the sample. The data from this sample is therefore not trustworthy.

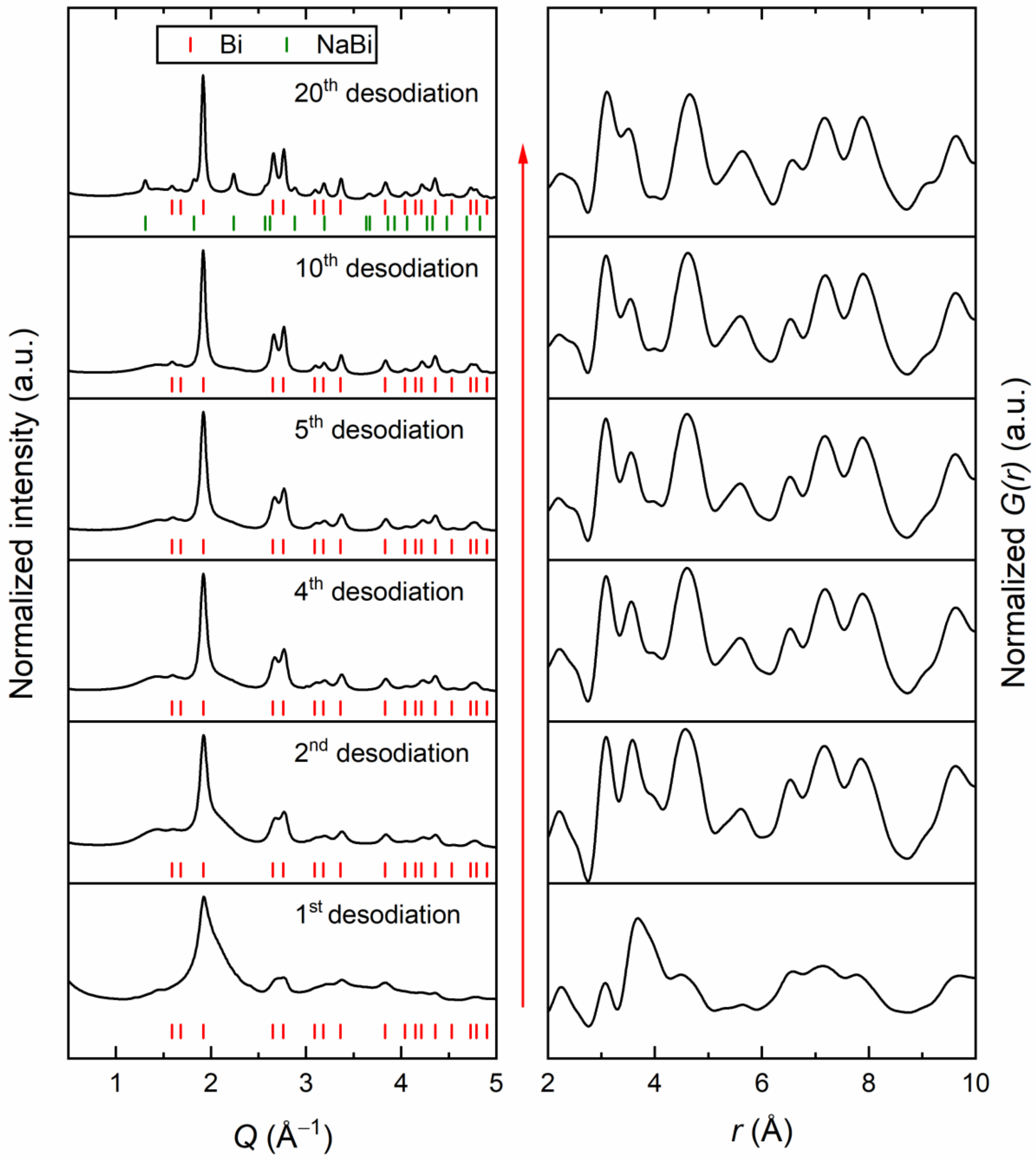

Figure S15: X-ray diffractograms (left) and corresponding PDFs (right) from ex situ measurements on Bi_2MoO_6 samples desodiated to 2.00 V vs Na/Na^+ from different cycles. The red arrow indicates the direction of cycling.

Section S8 – Ex Situ XANES and EXAFS on Bi L3 Edge

Ex situ XANES measurements of the Bi edge from samples extracted during the 1st sodiation show that Bi drastically changes oxidation state from +3 in Bi_2MoO_6, to negative oxidation states as the edge position moves towards lower energies and surpasses the Bi-foil reference at the fully sodiated state (Figure S16a). The oxidation state in the fully sodiated state is expected to be −3, since peaks corresponding to c-Na_3Bi are present in the corresponding diffractogram (Figure S11). However, because of the lack of reliable Bi^{3-} references, we are not able to directly prove it. In the corresponding FT EXAFS graphs, it is clear that the Bi−O

bonds and the more long-range signals corresponding to Bi−Bi and Bi−Mo distances in Bi_2MoO_6 disappear upon sodiation (Figure S16b). In the 0.80 V sample, there is a double peak between 2.0−3.3 Å similar to the Bi-metal reference (Figure S2b), corresponding to the closest Bi−Bi bonds. In the fully sodiated sample (0.01 V) the double peak has been shifted slightly and increased a bit in intensity indicating that we have formed Na−Bi bonds instead.

During the 1st desodiation Na_3Bi transforms back to Bi metal (samples at 0.70 and 1.00 V), as shown in Figure S16b. In the fully desodiated state (2.00 V), it is clear that Bi is more oxidized than Bi metal and we observe a clear peak corresponding to Bi−O bonds in the EXAFS FT between 1−2 Å (Figure S16c and d). In addition, a small shoulder on the right side of the peak at ~3.1 Å is observed, which could correspond to the Bi−Bi distances induced by oxidation of Bi that was observed in the PDF data (Section S7). Apart from this, there are no similarities to Bi_2O_3 and since we have Bi−Bi bonds and XRD peaks corresponding to Bi metal we most likely have Bi nanoparticles with Bi−O bonds at the interface between Bi and the Na−Mo−O matrix.

The system behaves similarly during the 2nd cycle (Figure S17) and the 5th cycle (Figure S18), but with gradually less oxidation of Bi. For the 5th cycle we also have samples representing the NaBi phase, which looks slightly different in the FT EXAFS graphs. When comparing the fully sodiated samples between 1st and 10th cycle, it is clear that they have the same features, which corresponds to c-Na_3Bi (Figure S19a and b). On the other hand, the sample from the 20th sodiation is significantly more oxidized than expected.[2] This proves that this sample reacted before the measurement as was indicated from the XRD data presented in Section S6.

For the fully desodiated samples (2.00 V) it is clear that Bi becomes less oxidized as a function of cycle number. After 1st and 2nd desodiation the XANES Bi edge position indicates an oxidation state above 0 for both samples, maybe as high as +2 for the 1st desodiation (Figure S19), and there are clear Bi−O interactions in the FT EXAFS graphs (Figure S19d). The desodiated samples from cycle 4, 5 and 10 show oxidation states only slightly higher than Bi metal and no clear Bi−O interactions in the FT EXAFS. This could be explained by the growth of the Bi nanoparticles (Section S7, and Figure 4 in the main article) leading to a reduction in the surface-to-volume ratio and therefore reducing the Bi−O interactions with the Na−Mo−O matrix. The desodiated sample from the 20th cycle has an average negative oxidation state for Bi (Figure S19c). We would expect the oxidation state to be even lower based on previous *operando* XRD data where the system consisted of a combination of Na_3Bi and NaBi in the desodiated state after 20 cycles.[2] Nevertheless, the data presented here proves that the system is not able to fully desodiate back to Bi and is partially stuck in the sodiated state after 20 cycles.

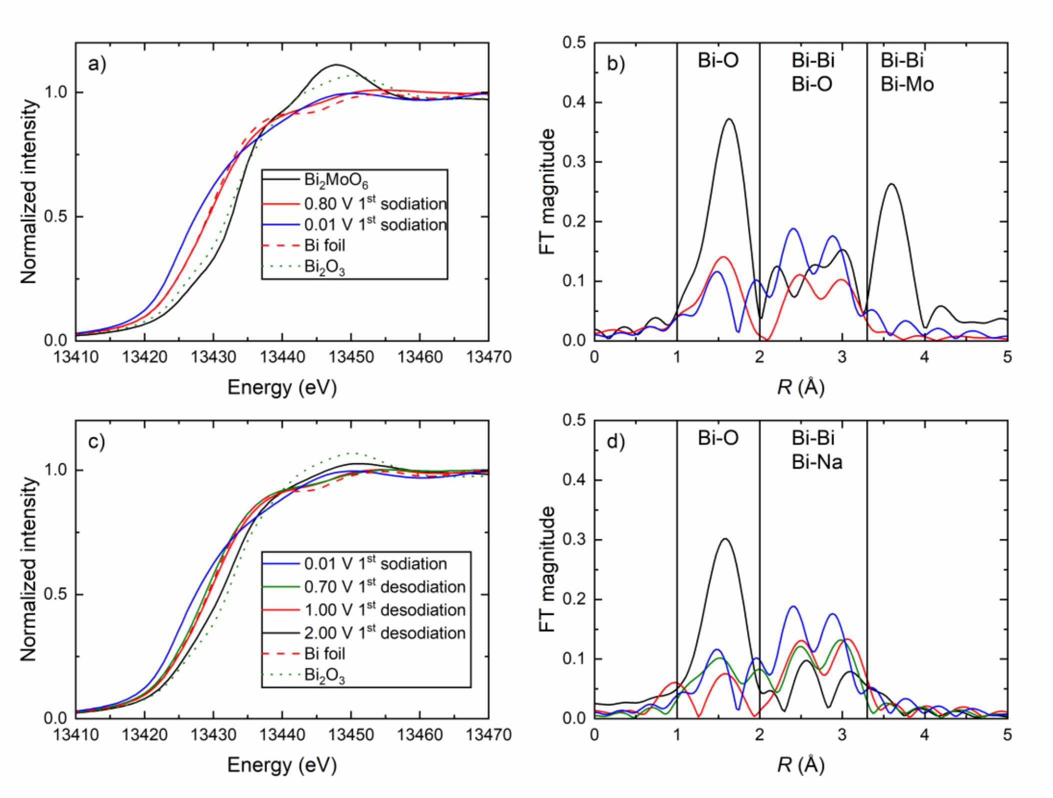

Figure S16: Results from ex situ XAS measurements on the Bi L3 edge from the 1st cycle of Bi_2MoO_6. a) XANES of the 1st sodiation, b) corresponding FT EXAFS graphs, c) XANES of the 1st desodiation and d) corresponding FT EXAFS graphs.

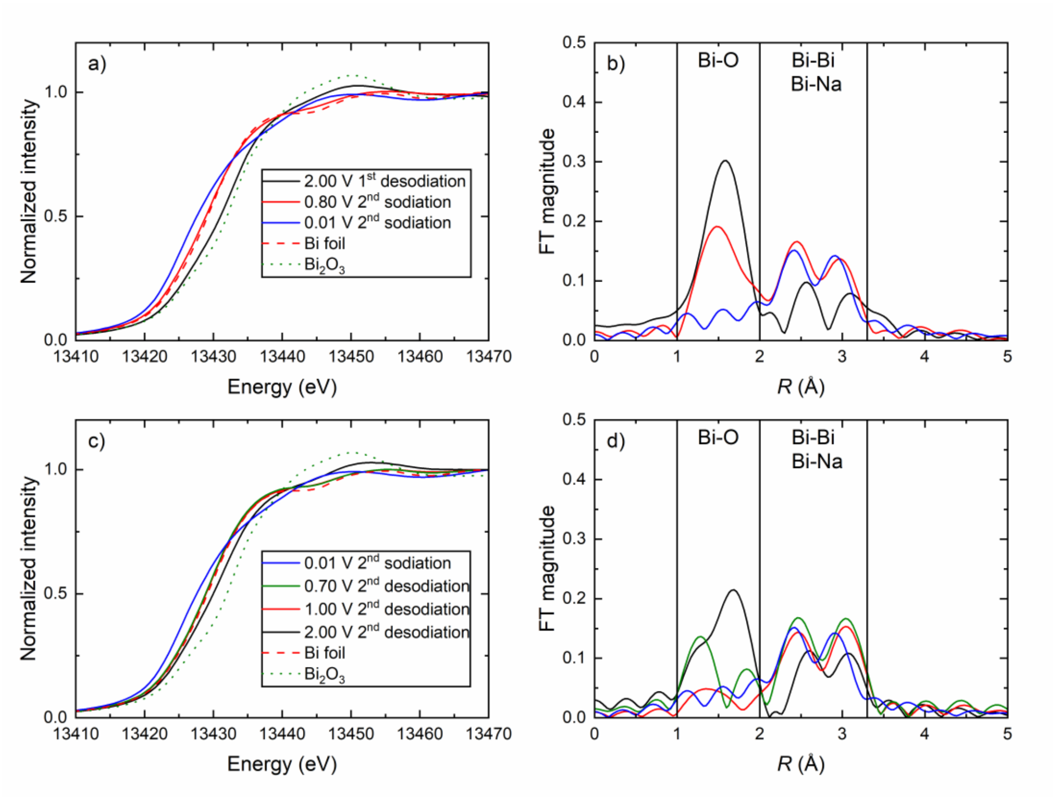

Figure S17: Results from ex situ XAS measurements on the Bi L3 edge from the 2nd cycle of Bi_2MoO_6. a) XANES of the 2nd sodiation, b) corresponding FT EXAFS graphs, c) XANES of the 2nd desodiation and d) corresponding FT EXAFS graphs.

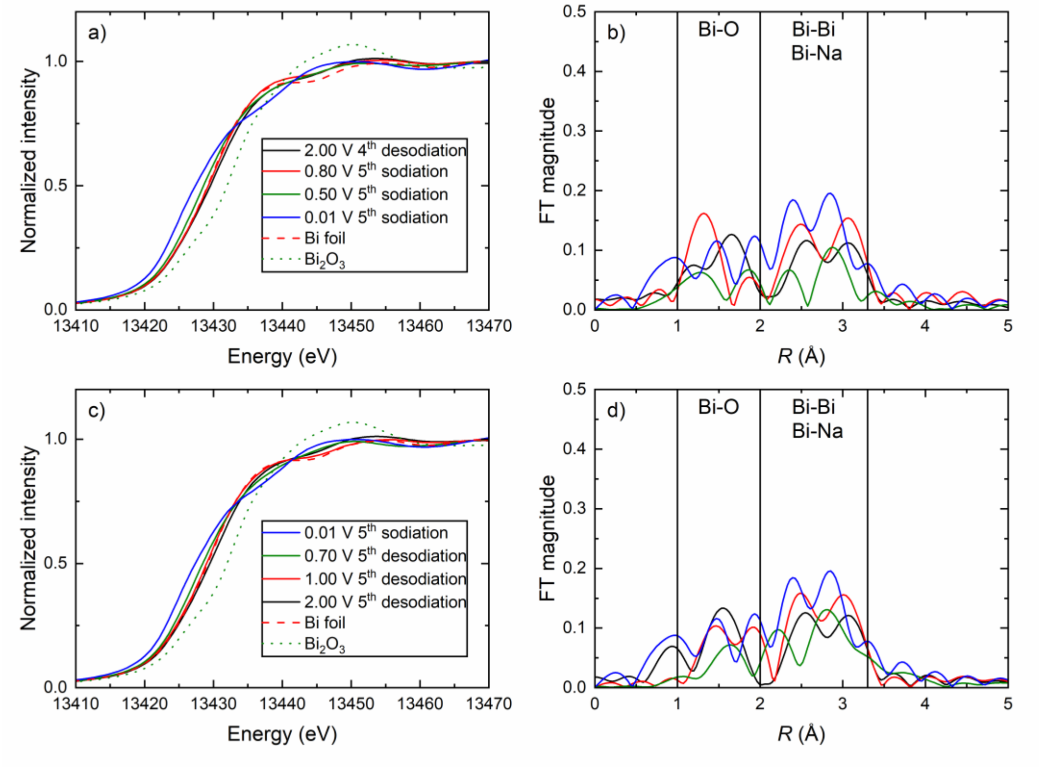

Figure S18: Results from ex situ XAS measurements on the Bi L3 edge from the 5th cycle of Bi_2MoO_6. a) XANES of the 5th sodiation, b) corresponding FT EXAFS graphs, c) XANES of the 5th desodiation and d) corresponding FT EXAFS graphs.

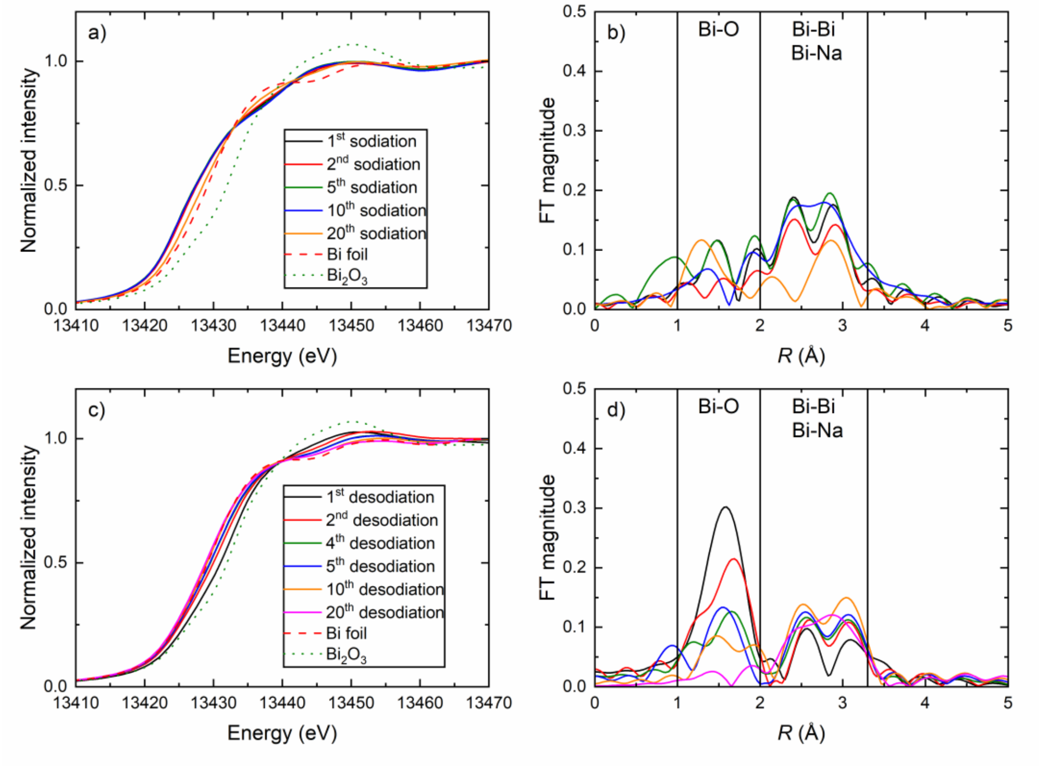

Figure S19: Results from ex situ XAS measurements on the Bi L3 edge of fully sodiated (0.01 V vs Na/Na^+) and desodiated (2.00 V vs Na/Na^+) samples of Bi_2MoO_6. a) XANES of the fully sodiated samples at different cycles, b) corresponding FT EXAFS graphs, c) XANES of the fully desodiated samples and d) corresponding FT EXAFS graphs.

Section S9 − Ex Situ XANES and EXAFS on Mo K Edge

From the XANES measurements in Figure S20−Figure S23, we do not observe any significant shift in the Mo edge, indicating that we have Mo^{6+} for all the samples, however, there is a clear change in the pre-edge. During the first sodiation the initial low pre-edge peak of the pristine material, corresponding to a distorted octahedral Mo−O coordination, increases in intensity during sodiation to 0.80 V as Mo^{6+} obtains tetrahedral coordination to O similar to that of Na_2MoO_4 (Figure S20a). After full sodiation to 0.01 V, the pre-edge has again decreased indicating a more octahedral coordination. This is also supported by the FT EXAFS where the Mo−O peak becomes taller, sharper and shift towards lower R for the more tetrahedral samples due to shorter, stronger and more defined bonds between Mo and O (Figure S20b). At higher R (3−4 Å), the peaks corresponding to Mo−Mo/Bi distances that are present for pristine Bi_2MoO_6 disappear because of the amorphization of the material, leaving almost all signals beyond the first coordination shell invisible. However, there is a peak emerging between 2−3 Å (Figure S20b), which could correspond to Na−Mo distances similar to those in Na_4MoO_5 (Section S10).

During the 1st desodiation the pre-edge gradually increases in intensity as Mo^{6+} transitions back to tetrahedral coordination from octahedral coordination (Figure S20c), accompanied by the sharpening of the Mo−O peak (Figure S20d). The possible Na−Mo peak also disappears and the data looks similar to Na_2MoO_4 except for the lack of long-range order. Therefore, it is likely that the Na−Mo−O matrix in the desodiated state is an amorphous Na_2MoO_4 phase.

The samples extracted from the 2nd cycle (Figure S21) and the 5th cycle (Figure S22) show coherent results with what was described for the 1st cycle. When comparing all the fully sodiated and desodiated samples from cycle 1, 2, 5, 10 and 20 it is clear that the sodiated samples have close to octahedrally coordinated Mo^{6+} with possible Na−Mo interactions. While the desodiated samples have tetrahedrally coordinated Mo^{6+} and a structure similar to Na_2MoO_4, except for the 20th desodiation, which is partially stuck in the sodiated state (as described in Section S7 and S8).

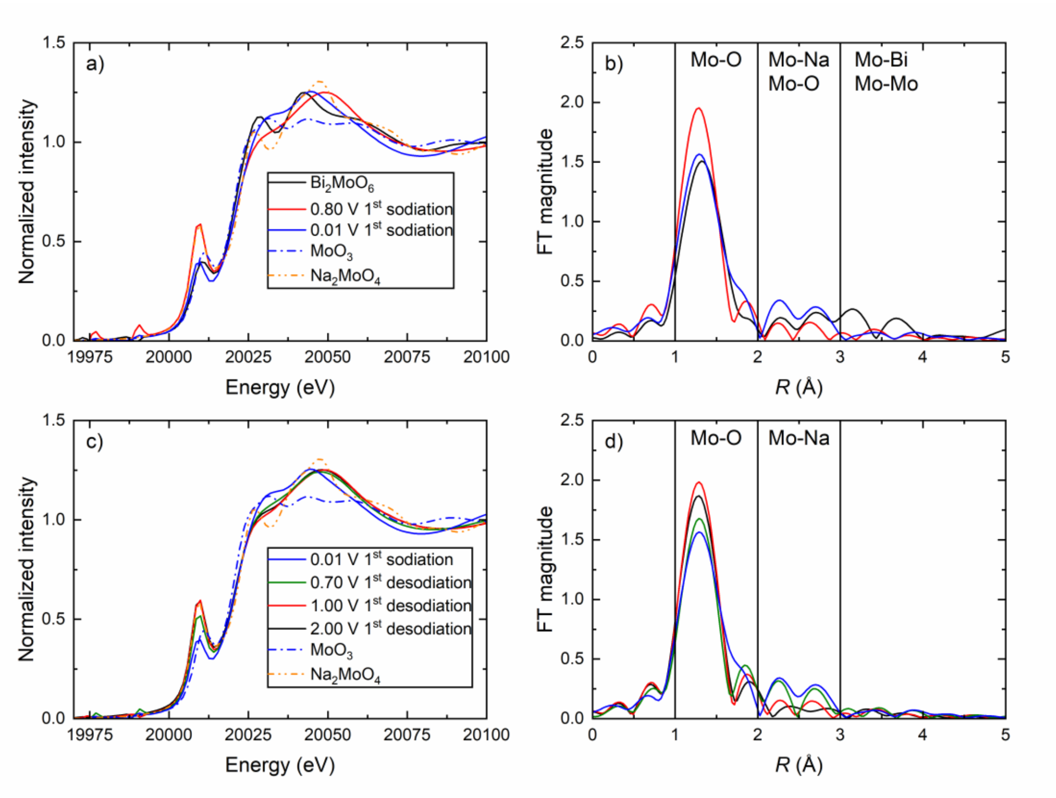

Figure S20: Results from ex situ XAS measurements on the Mo K edge from the 1st cycle of Bi_2MoO_6. a) XANES of the 1st sodiation, b) corresponding FT EXAFS graphs, c) XANES of the 1st desodiation and d) corresponding FT EXAFS graphs.

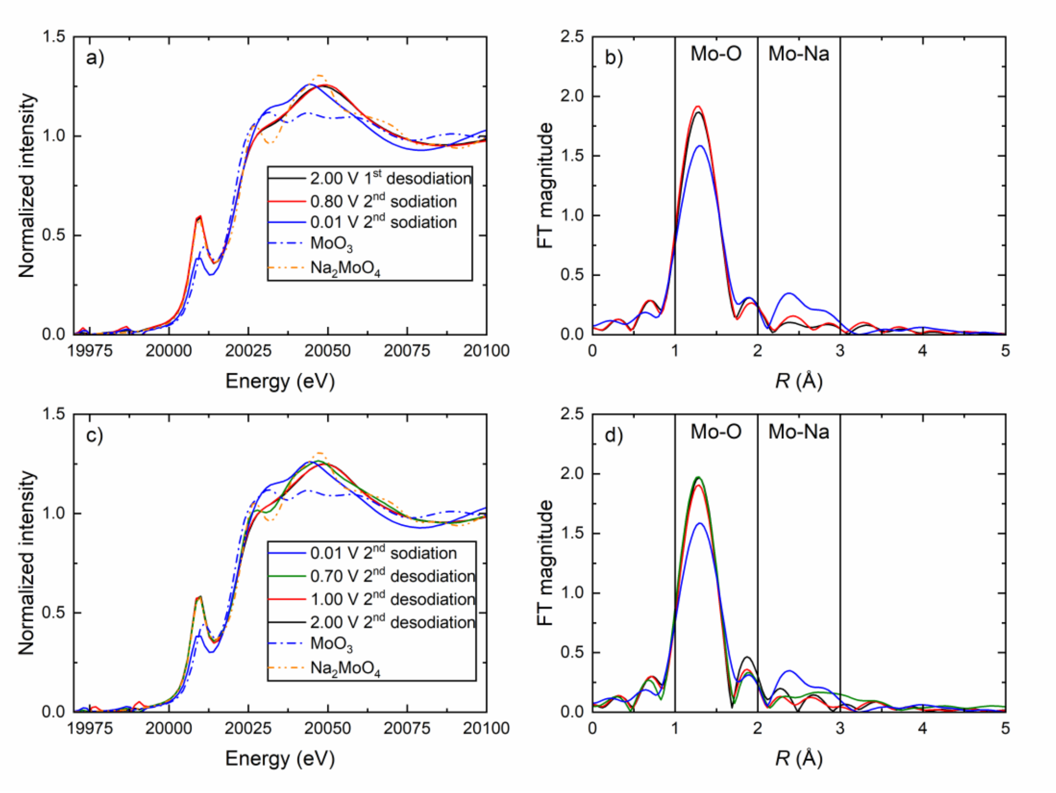

Figure S21: Results from ex situ XAS measurements on the Mo K edge from the 2nd cycle of Bi_2MoO_6. a) XANES of the 2nd sodiation, b) corresponding FT EXAFS graphs, c) XANES of the 2nd desodiation and d) corresponding FT EXAFS graphs.

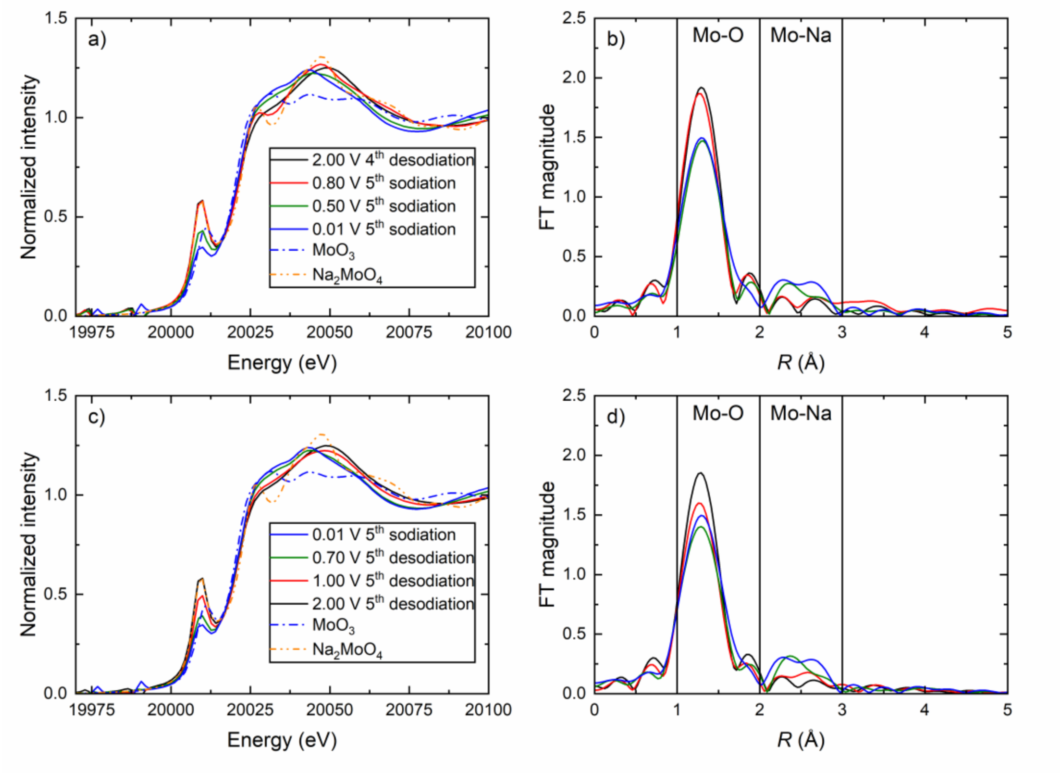

Figure S22: Results from ex situ XAS measurements on the Mo K edge from the 5th cycle of Bi_2MoO_6. a) XANES of the 5th sodiation, b) corresponding FT EXAFS graphs, c) XANES of the 5th desodiation and d) corresponding FT EXAFS graphs.

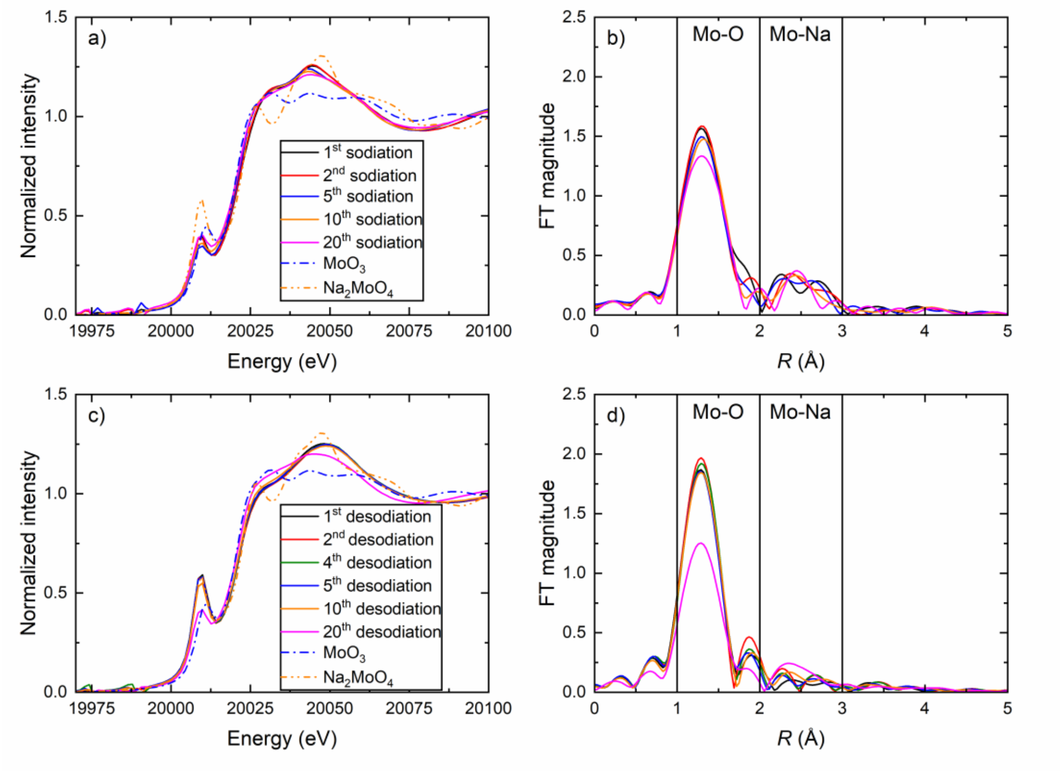

Figure S23: Results from ex situ XAS measurements on the Mo K edge from fully sodiated (0.01 V vs Na/Na^+) and desodiated (2.00 V vs Na/Na^+) samples of Bi_2MoO_6. a) XANES of the fully sodiated samples at different cycles, b) corresponding FT EXAFS graphs, c) XANES of the fully desodiated samples and d) corresponding FT EXAFS graphs.

Section S10 – Fitting of Sodiated Bi_2MoO_6

Two Mo−O bonds and two Na−Mo bonds from the Na_4MoO_5 crystal structure (COD: 1534864) were used to fit the FT EXAFS graph of a Bi_2MoO_6 sample after 1st sodiation in Artemis (Figure S24).[8] Given the apparent amorphous structure of the Na−Mo−O matrix, it is likely that there are many different short Mo−O and Na−Mo distances. However, including too many distances in the fit would likely provide a close to perfect fit no matter what the data looks like, which could be counterproductive. Therefore, a limited selection of two Mo−O bonds (from the Na_4MoO_5 phase) was chosen to account for the broad Mo−O peak, and two Na−Mo distances were chosen to account for the double peak between 2−3 Å. The Mo−O bonds also influence the 2−3 Å region, which is why they had to be included and why we could not only fit the 2−3 Å region. The calculated bond distances in Table S3 are not meant as exact values of well-defined bonds, but as an indication of the range of Mo−O and Na−Mo distances that we could expect to have in the sample. This result indicates that there are several Mo−O bonds with distances of 1.8−2.0 Å and Na−Mo bonds with distances between 2.8−3.3 Å in the sample.

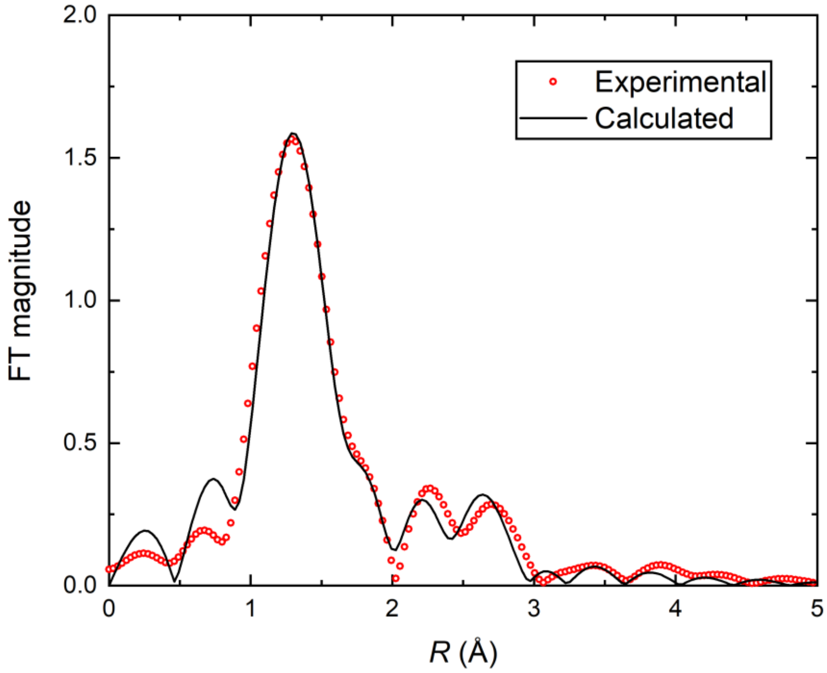

Figure S24: Experimental vs calculated data from fitting of FT EXAFS data of Bi_2MoO_6 after the 1st sodiation, using two Mo−O and two Na−Mo interactions selected from the Na_4MoO_5 structure.

Table S3: Bond distances estimated from fitting in Figure S24.

Atomic bonds	Selected distances from Na_4MoO_5 (Å)	Calculated distances (Å)
Mo−O	1.76	1.80
Mo−O	1.95	1.96
Na−Mo	3.04	2.89
Na−Mo	3.26	3.25

References:

(1) Coelho, A. A. TOPAS and TOPAS-Academic: An Optimization Program Integrating Computer Algebra and Crystallographic Objects Written in C++. *J. Appl. Crystallogr.* **2018**, *51* (1), 210-218.
(2) Brennhagen, A.; Cavallo, C.; Wragg, D. S.; Vajeeston, P.; Sjåstad, A. O.; Koposov, A. Y.; Fjellvåg, H. Operando XRD Studies on Bi_2MoO_6 as Anode Material for Na-ion Batteries. *Nanotechnology* **2022**, *33* (18), 185402.
(3) Sottmann, J.; Herrmann, M.; Vajeeston, P.; Hu, Y.; Ruud, A.; Drathen, C.; Emerich, H.; Fjellvåg, H.; Wragg, D. S. How Crystallite Size Controls the Reaction Path in Nonaqueous Metal Ion Batteries: The Example of Sodium Bismuth Alloying. *Chem. Mater.* **2016**, *28* (8), 2750-2756.
(4) Brennhagen, A.; Skautvedt, C.; Cavallo, C.; Wragg, D.; Koposov, A. Y.; Sjåstad, A. O.; Fjellvåg, H. Unraveling the (De)Sodiation Mechanisms of $BiFeO_3$ at a High Rate with *Operando* XRD. *ACS Apppl. Mater. Interfaces* **2024**, *16(10)*, 12428-12436.
(5) Brennhagen, A.; Nafuma. Experimental Data for the Nanoname Project, DataverseNO, 2023, https://doi.org/10.18710/ZFADJS.
(6) Liu, C.-H.; Wright, C. J.; Gu, R.; Bandi, S.; Wustrow, A.; Todd, P. K.; O'Nolan, D.; Beauvais, M. L.; Neilson, J. R.; Chupas, P. J. Validation of Non-Negative Matrix Factorization for Rapid Assessment of Large Sets of Atomic Pair Distribution Function Data. *J. Appl. Crystallogr.* **2021**, *54* (3), 768-775.
(7) Thatcher, Z.; Liu, C.-H.; Yang, L.; McBride, B. C.; Thinh Tran, G.; Wustrow, A.; Karlsen, M. A.; Neilson, J. R.; Ravnsbæk, D. B.; Billinge, S. J. nmfMapping: a Cloud-Based Web Application for Non-Negative Matrix Factorization of Powder Diffraction and Pair Distribution Function Datasets. *Acta Crystallogr. Sect. A: Found. Adv.* **2022**, *78* (3), 242-248.
(8) Ravel, B.; Newville, M. ATHENA, ARTEMIS, HEPHAESTUS: Data Analysis for X-Ray Absorption Spectroscopy using IFEFFIT. *J. Synchrotron Radiat.* **2005**, *12* (4), 537-541.
